# Decoding sex and gender effects on health: evidence from a nationwide cohort

**DOI:** 10.1186/s13293-026-00888-8

**Published:** 2026-03-30

**Authors:** Caroline E. Gebhard, Bianca Gysi, Pimrapat Gebert, Nidaa Mikail, Livia Liechti, Patrick R. Schmidlin, Adriana Vinzens, Achi Haider, Susan Bengs, Valerie Treyer, Philipp K. Buehler, Reto A. Schuepbach, Annelies S. Zinkernagel, Silvio D. Brugger, Dimitri Patriki, Benedikt Wiggli, Jürg H. Beer, Andrée Friedl, Raphael Twerenbold, Gabriela M. Kuster, Joerg C. Schefold, Thibaud Spinetti, Pedro D. Wendel-Garcia, Daniel A. Hofmaenner, Thomas Scheier, Martin Siegemund, Vera Regitz-Zagrosek, Catherine Gebhard

**Affiliations:** 1 Intensive Care Unit, Department of Acute Medicine, University Hospital Basel, Petersgraben 4, 4031 Basel, Switzerland; 2 University of Basel, Basel, Switzerland; 3 Institute of Biometry and Clinical Epidemiology, Charité, Universitätsmedizin Berlin, Berlin, Germany; 4 Department of Nuclear Medicine, University Hospital Zurich, University of Zurich, Zurich, Switzerland; 5 Center for Molecular Cardiology, University of Zurich, Schlieren, Switzerland; 6 Clinic of Conservative and Preventive Dentistry, Center of Dental Medicine, University of Zurich, Zurich, Switzerland; 7 Pharma Research and Early Development, Roche Innovation Center Basel, F. Hoffmann-La Roche Ltd, Basel, Switzerland; 8 Institute of Intensive Care, University Hospital Zurich, University of Zurich, Zurich, Switzerland; 9 Institute of Intensive Care Medicine, Cantonal Hospital Winterthur, Winterthur, Switzerland; 10 Department of Infectious Diseases and Hospital Epidemiology, University Hospital Zurich, University of Zurich, Zurich, Switzerland; 11 Department of Cardiology, University Hospital Zurich, University of Zurich, Zurich, Switzerland; 12 Infectious Diseases and Hospital Epidemiology, Department of Internal Medicine, Cantonal Hospital of Baden, Baden, Switzerland; 13 Department of Internal Medicine, Cantonal Hospital of Baden, Baden, Switzerland; 14 Department of Cardiology, University Hospital Basel, Basel, Switzerland; 15 Department of Cardiology and University Center of Cardiovascular Science, University Heart and Vascular Center Hamburg, Hamburg, Germany; 16 Department of Biomedicine, University of Basel and University Hospital Basel, Basel, Switzerland; 17 Department of Intensive Care Medicine, Inselspital Bern University Hospital, University of Bern, Bern, Switzerland; 18 Charité, Universitätsmedizin Berlin, Berlin, Germany; 19 University of Zurich, Zurich, Switzerland; 20 Department of Cardiology, Bern University Hospital Inselspital, University of Bern, Bern, Switzerland

**Keywords:** Cardiometabolic risk, Sex, Gender, Sociocultural

## Abstract

**Background:**

Validated tools to assess gender are essential for clinical and population research but remain scarce and unstandardized. Understanding how sociocultural gender interacts with biological sex in shaping disease risk is critical for precision medicine. We aimed to investigate the association of sex and gender with cardiovascular risk factors and common comorbidities, and to refine an existing composite Gender score in a Swiss cohort.

**Methods:**

This multicenter prospective study conducted in three tertiary and one regional hospital included outpatients and inpatients with PCR-confirmed SARS-CoV-2 infection (n = 2,690; estimation and internal validation sets) and outpatients with periodontitis (n = 337; external validation set). Gender-related variables were acquired via questionnaire. Logistic regression models and propensity score matching were used to assess associations between sex, gender, and the prevalence of cardiometabolic and chronic health conditions.

**Results:**

A total of 3,027 individuals (46.3% women; mean age 45 ± 17 years) were included. The composite Gender Score predicted biological sex with good accuracy (ROC 0.776–0.809). Biological sex was more strongly associated with most cardiometabolic risk factors, stroke, immune disorders, and bone diseases, whereas gender showed limited independent associations. For diabetes, female sex was inversely associated with diabetes prevalence, while a more feminine gender profile was positively associated. These opposing associations persisted after accounting for gender in matched analyses, illustrating that sex and gender may contribute differently to specific metabolic outcomes.

**Conclusion:**

Sociocultural variables can accurately approximate biological sex and are differentially associated with health outcomes. In this cohort, biological sex explained most associations with cardiometabolic and chronic conditions, while gender effects were modest and condition-specific. The divergent associations observed for diabetes highlight that sex and gender are related but distinct constructs that should be considered jointly. Integrating both dimensions may improve future approaches to risk stratification, although these findings require confirmation in prospective and diverse populations.

**Supplementary Information:**

The online version contains supplementary material available at 10.1186/s13293-026-00888-8.

## Highlights

A composite Gender Score, based on less than 7 sociocultural items, accurately approximated biological sex in a Swiss cohort.

Biological sex explained most associations with cardiometabolic and chronic health conditions.

Gender showed limited independent associations, with outcome-specific effects.

Opposing sex–gender associations were most evident for diabetes risk.

Disentangling sex and gender helps clarify when sociocultural factors add information beyond sex.

**Supplementary Information:**

The online version contains supplementary material available at 10.1186/s13293-026-00888-8.

## Abstract (plain language summary)

Men and women often differ in their health not only because of biological factors, but also because of social roles and life circumstances. Biological sex refers to physical differences between males and females, while gender describes social and cultural factors such as work roles, caregiving responsibilities, income, and stress.

In this study, we developed and tested a “Gender Score” based on social and behavioral characteristics in more than 3,000 people in Switzerland. We then examined how biological sex and sociocultural gender were related to common health conditions, including heart-related, metabolic, immune, and infectious diseases.

We found that biological sex was more strongly linked to most health conditions than gender. In this Swiss population, being female or male explained variation in cardiometabolic and chronic health conditions, while gender-related factors showed fewer and more specific associations. One notable exception was diabetes: being biologically female was linked to a lower risk, while people with more traditionally feminine social characteristics - such as higher caregiving demands and stress - had a higher risk, regardless of sex.

These findings suggest that sex and gender are related but not the same, and they can influence health in different ways. In relatively gender-equal societies, social gender factors may play a smaller role overall, but they can still be important for specific conditions. Considering both biology and social context may help improve future health research and risk assessment, especially when studied over time.

**Supplementary Information:**

The online version contains supplementary material available at 10.1186/s13293-026-00888-8.

## Introduction

Sex and gender are increasingly recognized as distinct but complementary determinants of health. While sex refers to biological attributes such as chromosomes, reproductive anatomy, and hormone profiles, gender encompasses sociocultural roles, behaviors, and identities that vary across time and context. Briefly, gender encompasses multiple interrelated dimensions, including gender roles (e.g., childcare responsibilities), gender identity (self-perception as male, female, or non-binary), gender relationships (e.g., social support), and institutionalized gender (e.g., education level) [1]. Despite the lack of a universally accepted definition of "gender," it is increasingly recognized as a key determinant of health, influencing disease processes as significantly as age, ethnicity, or comorbidities [2, 3]. However, gender considerations remain largely absent from risk prediction tools, and the interplay between sex and gender in shaping disease traits is poorly understood.

Growing evidence indicates that sex and gender jointly influence the onset and progression of disease. In cardiometabolic health, for example, biological sex appears to favor women, with men typically developing atherosclerotic disease earlier [1, 4]. However, women often experience worse outcomes after cardiovascular events, suggesting gender-related factors—such as help-seeking behavior, physical activity, diet, and exposure to stress—further shape individual risk profiles and contribute to inequalities in care. Intersecting biological and sociocultural influences are particularly evident in cardiovascular disease, where gender norms can limit access to timely diagnosis and appropriate treatment, especially for women [1, 4].

Despite mounting evidence for the relevance of gender in health, most population-based studies still rely exclusively on binary sex variables. This limited approach risks overlooking key dimensions of vulnerability and resilience that emerge from social context. To address this gap, we and others have developed Gender Scores, which distill key gender-related variables into a single composite measure for use in risk prediction models—akin to biological sex [5–10]. This approach enhances statistical power and simplifies interpretation compared to analyzing multiple individual gender-related variables. Gender scores typically include sociocultural factors historically differing between men and women, such as education, parental and marital status, income, household and childcare responsibilities, stress levels at home, and self-reported gender-related traits (e.g., from the BEM Sex-Role Inventory) [11, 12]. A recently developed abbreviated gender score was applied in a Swiss cohort of 3,000 SARS-CoV-2-positive individuals [9, 10]. While male sex was a stronger predictor of acute COVID-19 severity [9], sociocultural gender factors were key predictors of Post-COVID-19 Syndrome, which is more prevalent in women [10]. However, in this study, individual gender-related variables, rather than the overall gender score, were the strongest predictors of long-term outcomes after SARS-CoV-2 infection [10].

Accordingly, we aimed to examine which sex- and gender-related variables are associated with cardiometabolic and other chronic health conditions beyond COVID-19 in a Swiss population, and to refine the existing composite Gender Score by identifying the most informative sociocultural variables distinguishing women and men.

## Methods

### Study design, procedures, and data sources

This study analyzed data from the Swiss COGEN cohort, a prospective observational study of individuals with PCR-confirmed SARS-CoV-2 infection diagnosed between February and December 2020 at four Swiss hospitals: University Hospital Basel, University Hospital Zurich, University Hospital Bern, and Cantonal Hospital Baden. Cohort details have been previously described [9, 10] Eligible participants were adults (≥ 18 years) who survived acute COVID-19, resided in Switzerland during their primary infection, and were fluent in German, English, French, or Italian. All individuals provided written informed consent. Participants completed a questionnaire via phone, email, or in written form, covering education, parental and marital status, income, household and childcare responsibilities, stress levels at home, and traits historically labelled as feminine/masculine assessed via the BEM Sex-Role Inventory. The BEM items reflect culturally defined gender role characteristics traditionally labelled as ‘masculine’ or ‘feminine’; in this study, we use these terms descriptively, based on self-reported traits, without implying biological determinism or reinforcing normative gender stereotypes. Supplementary Fig. 1 lists the gender-related items used in the questionnaire, including the BEM traits and sociocultural variables. Of 5,938 eligible patients, 3,005 completed the questionnaire, including nine via legal representatives. After excluding 315 cases with missing gender-related data, the final analysis included 2,690 participants. Supplementary Fig. 2 shows the flowchart of the estimation and validation datasets used in our analysis. To evaluate the predictive performance of the gender score model, the cohort was randomly split into an estimation set (1,882 participants, 70%) used for model development and an internal validation set (808 participants, 30%) used for performance testing (Supplementary Fig. 2). Clinical and laboratory data—including demographic details, medication use, and physician-diagnosed cardiovascular and chronic conditions—were extracted from electronic medical records and complemented by patient-reported questionnaires. For external validation, a separate cohort of 337 outpatients with suspected periodontitis at the University of Zurich, recruited between May 2021 and October 2022, completed the same questionnaire on the day of consultation (Supplementary Fig. 2). The external dataset was used solely to validate the stability and applicability of the Gender Score model in an independent, demographically distinct population. It was not intended for replication of the associations with cardiometabolic and chronic health conditions. Ethical approval was obtained from the Ethics Committees of Northwest and Central Switzerland (EKNZ, BASEC #2020–01311) and Zurich (BASEC #2020–02864). Patient recruitment and exclusions are detailed in the flowchart (Supplementary Fig. 2).

### Outcome definitions

Prevalence of cardiometabolic and chronic health conditions was determined using both structured patient questionnaires and data from electronic medical records. Participants reported their medical conditions and medications, and these self-reports were verified against medical records when available. Conditions were defined as follows: diabetes by physician diagnosis, antidiabetic medication, or documented hyperglycemia; hypertension by diagnosis or use of antihypertensive drugs; dyslipidemia by diagnosis or lipid-lowering therapy; and obesity by a body mass index ≥ 30 kg/m2. Coronary artery disease (CAD) and stroke or transient ischemic attack (TIA) were based on documented history or relevant interventions. Immune system disorders included physician-diagnosed chronic autoimmune or inflammatory diseases (e.g., lupus, rheumatoid arthritis). Psychiatric diseases encompassed mood, anxiety, bipolar, or psychotic disorders as reported or documented, as well as the use of antidepressant medication. Pulmonary diseases included asthma, chronic obstructive pulmonary disease (COPD), and pulmonary hypertension. Bone diseases were primarily osteopenia and osteoporosis confirmed by bone mineral density or clinical diagnosis. Infectious diseases included chronic infections such as HIV or hepatitis. Chronic pain disorders were longstanding pain syndromes such as fibromyalgia, chronic back pain, or neuropathic pain. Gastrointestinal diseases included chronic conditions affecting the stomach or intestines, such as inflammatory bowel disease (e.g., Crohn’s disease, ulcerative colitis), irritable bowel syndrome, or chronic gastritis, as reported by the patient or documented in medical records. There were no missing values for prevalence of cardiometabolic and chronic health conditions, as participants with incomplete medical histories or missing gender-related variables were excluded from the analysis.

### Study questionnaire and gender assessment

Data were collected using the Research Electronic Data Capture (REDCap) system. Gender is a multifaceted concept encompassing four key dimensions: gender roles (e.g., caregiving responsibilities), gender identity (self-identification as male, female, or other), gender relationships (e.g., social support), and institutionalized gender (e.g., education and income levels) [13, 14]. While various frameworks exist to define and measure gender, no universally accepted standard has been established [2]. A widely used approach introduced by Pelletier et al. applies a composite gender score, ranging from 0 (traditionally masculine traits, attitudes, and behaviors) to 100 (traditionally feminine traits) [5]. This method integrates multiple gender-related variables and has been successfully used to assess gender’s impact on health outcomes. Its advantage lies in treating gender as a continuum, allowing for a single-variable statistical model, which enhances statistical power and simplifies interpretation. For this study, we used a short-form questionnaire (Supplementary Fig. 1) including items on employment status, household responsibilities, education, social support, domestic stress, and selected questions from the BEM Sex-Role Inventory [5, 7, 15]. This short-form score has performed as well as the full version in Canadian and German cohorts and was chosen due to its successful application in previous Swiss studies [5, 7]. The BEM items reflect culturally defined gender role characteristics traditionally labelled as ‘masculine’ or ‘feminine’. In line with the original BEM framework, these terms describe sociocultural traits along a continuum rather than fixed or biologically determined attributes. In this study, they are used descriptively to capture self-reported gender-related roles and behaviors within a specific sociocultural context.

### Implementing the gender score model

In the estimation dataset (n = 1,882), we first assessed correlations among gender-related variables, confirming the absence of multicollinearity (no correlation r^2^ > 0.8). Principal component analysis (PCA) was then performed to reduce the number of variables by identifying the most relevant ones for this population. To determine which gender-related variables best predicted biological sex, we applied a logistic regression model using a backward-stepwise approach, sequentially removing non-significant variables (p > 0.05) [5]. The final model was selected based on the lowest Akaike Information Criterion (AIC) and Bayesian Information Criterion (BIC) values. The gender score was derived from the logistic regression coefficients, as previously described, and represents the predicted probability of being female [5, 7]. Scores range from 0 (predominantly traits historically labelled as masculine) to 100 (predominantly traits historically labelled as feminine).

### Statistical analyses

Descriptive statistics were presented separately for the three datasets. Continuous socioeconomic characteristics were compared between biological sexes using independent t-tests, and among datasets and gender score tertiles using one-way ANOVA. Categorical variables were analyzed using chi-square tests. Logistic regression models assessed associations between biological sex, gender score, and cardiometabolic and chronic health conditions. Gender was included either as a single continuous variable (Gender Score) or as multiple variables representing its components.

To assess the associations between sex, gender, and cardiometabolic and chronic health conditions, we applied logistic regression models adjusted for age, which was retained as a covariate because it is a dominant confounder across all outcomes. Gender was operationalized both as a continuous variable (Gender Score) and as tertiles (low, middle, high), with the middle tertile used as the reference category. We first conducted age-adjusted models evaluating the association of biological sex with cardiometabolic and chronic health conditions without adjustment for gender, as well as models evaluating the association of gender tertiles with disease outcomes without adjustment for sex. We then constructed fully adjusted models including age, biological sex, and gender tertiles to assess their independent associations with cardiometabolic and chronic health conditions. To isolate the effect of sex independent of gender, we applied propensity score matching using a nearest-neighbor algorithm [12]. Men and women were matched on Gender Score until no further pairs could be formed, discarding pairs with gender score differences exceeding 0.2 standard deviations [16]. Since matched pairs had similar gender characteristics, any remaining differences were attributed to biological sex. Conditional logistic regression models were then used to examine associations between biological sex and cardiometabolic and chronic health conditions post-matching. All models were adjusted for age, and results were reported as adjusted odds ratios (OR) with 95% confidence intervals (CI). Statistical analyses followed an exploratory framework with a two-sided significance level of α = 0.05 and were conducted using Stata 18/MP (StataCorp, 2023, College Station, TX, USA).

## Results

### Baseline characteristics of the study cohorts

We compared demographic and sociocultural characteristics across the estimation, internal validation, and external validation cohorts. The estimation and internal validation cohorts had similar age and sex distributions, while the external validation cohort was significantly older (44 ± 18 and 43 ± 17 years vs. 60 ± 12 years) and had a higher proportion of females (45.0% and 45.4% vs. 53.1%, Table 1). As detailed in Supplementary Table 1, which provides sex-stratified socioeconomic characteristics across all cohorts, no significant differences were observed between cohorts in marital status, income, household and childcare responsibilities, domestic stress levels, or most BEM questions.

**Table 1:** Socioeconomic characteristics of the study population

Sociocultural-and economic variables	Estimation set (n = 1,882)	Internal validation set (n = 808)	External validation set (n = 337)	p-value
Sex				0.029
Male (%)	1,026 (54.5)	441 (54.6)	158 (46.9)	
Female (%)	856 (45.5)	367 (45.4)	179 (53.1)	
Age (years) – mean (SD)	43.3 (16.5)	44.4 (17.6)	59.5 (11.7)	< 0.001
Education				< 0.001
No education qualification (%)	111 (5.9)	39 (4.8)	11 (3.3)	
Primary education (%)	114 (6.1)	56 (6.9)	26 (7.7)	
Secondary education or vocational degree (%)	735 (39.1)	324 (40.1)	187 (55.5)	
University or technical college degree (%)	922 (49.0)	389 (48.1)	113 (33.5)	
Marital status				0.26
Married/partnership (%)	1,272 (67.6)	560 (69.3)	242 (71.8)	
Living alone (%)	610 (32.4)	248 (30.7)	95 (28.2)	
Income				0.16
Earns highest income in household (%)	1,082 (57.5)	438 (54.2)	194 (57.6)	
Earns lowest income in household (%)	515 (27.4)	239 (29.6)	80 (23.7)	
Equal between partners (%)	285 (15.1)	131 (16.2)	63 (18.7)	
Main person responsible for household work				0.17
No (%)	442 (23.5)	198 (24.5)	61 (18.1)	
Yes (%)	799 (42.5)	332 (41.1)	158 (46.9)	
Equal distribution between partners	641 (34.1)	278 (34.4)	118 (35.0)	
Average domestic stress level (score 0—10) – Mean (SD)	3.3 (2.2)	3.4 (2.2)	3.1 (2.1)	0.059
Main responsibility for childcare/care of family members (min 1—max 6) – mean (SD)	1.7 (2.2)	1.8 (2.2)	1.6 (2.0)	0.31
BEM score	4.9 (0.9)	4.9 (0.9)	4.9 (0.9)	0.69
I am someone who … (min 1 – max 7) – mean (SD)				
…defends own opinion	5.5 (1.4)	5.5 (1.4)	5.4 (1.3)	0.56
…has leadership qualities	5.0 (1.5)	4.9 (1.5)	5.0 (1.4)	0.38
…is independent	5.7 (1.4)	5.7 (1.4)	5.6 (1.3)	0.81
…is willing to take risks	4.4 (1.6)	4.4 (1.6)	4.6 (1.5)	0.15
…is positive	5.5 (1.4)	5.5 (1.4)	4.7 (1.6)	< 0.001
…is assertive	5.2 (1.3)	5.2 (1.4)	5.3 (1.2)	0.62
…has a strong personality	5.4 (1.3)	5.4 (1.3)	5.3 (1.2)	0.78
…is ready to take a stand	5.4 (1.3)	5.5 (1.3)	5.6 (1.2)	0.28
…is energetic	4.8 (1.5)	4.8 (1.5)	4.9 (1.4)	0.70
…is aggressive	2.4 (1.4)	2.5 (1.5)	2.7 (1.5)	0.046
Self-assessment of gender identity (scale 1–7; 1 = predominantly traits traditionally labelled as masculine, 7 = predominantly traits traditionally labelled as feminine), mean (SD)	3.8 (2.1)	3.8 (2.1)	3.9 (2.2)	0.72

The study cohort was split into an estimation set (n = 1882) and an internal validation set (n = 808). An external validation set (n = 337) derived from a different population and time period was used to assess the generalizability and robustness of our predictive model. SD, standard deviation; BEM score, measure used to assess gender roles

### Sex-stratified sociocultural characteristics

We examined how key sociocultural variables differed between biological sexes across cohorts. Across all cohorts, females were more likely to have lower household incomes, take primary responsibility for household tasks, and report higher domestic stress levels (Supplementary Table 1). They also scored lower on the BEM items "I am someone who has leadership qualities" and "I am someone who is aggressive." In the internal validation cohort, females were more likely to live alone (34.6% vs. 27.4%) and take primary responsibility for childcare, though these differences were not observed in the other cohorts. Self-perceived gender differed significantly by sex in all cohorts, with females more often attributing feminine traits to themselves and males exhibiting the opposite pattern (Supplementary Table 1).

### Principal component analysis of gender-related variables

PCA was used to identify uncorrelated components that captured key dimensions of gender-related sociocultural traits. Before conducting PCA, we assessed correlations among gender-related variables in the estimation cohort (n = 1,882) and found no variables with a correlation coefficient above 0.8, allowing all variables to be included. Supplementary Table 2 presents the correlation coefficients confirming absence of multicollinearity. PCA, performed on the same cohort, identified 10 uncorrelated principal components (PCs, Supplementary Table 3, which lists the factor loadings and variance explained by each PC), explaining 83.8% of the total variance. The most influential contributors included household responsibility (PC9), education (PC7), income (PC8), domestic stress (PC5), marital status (PC6), and childcare or family care responsibilities (PC10), along with four BEM variables (PC1–4). The first PC captured the largest variance, with subsequent PCs contributing progressively less.

### Distribution of the gender score across cohorts

**Fig. 1:**
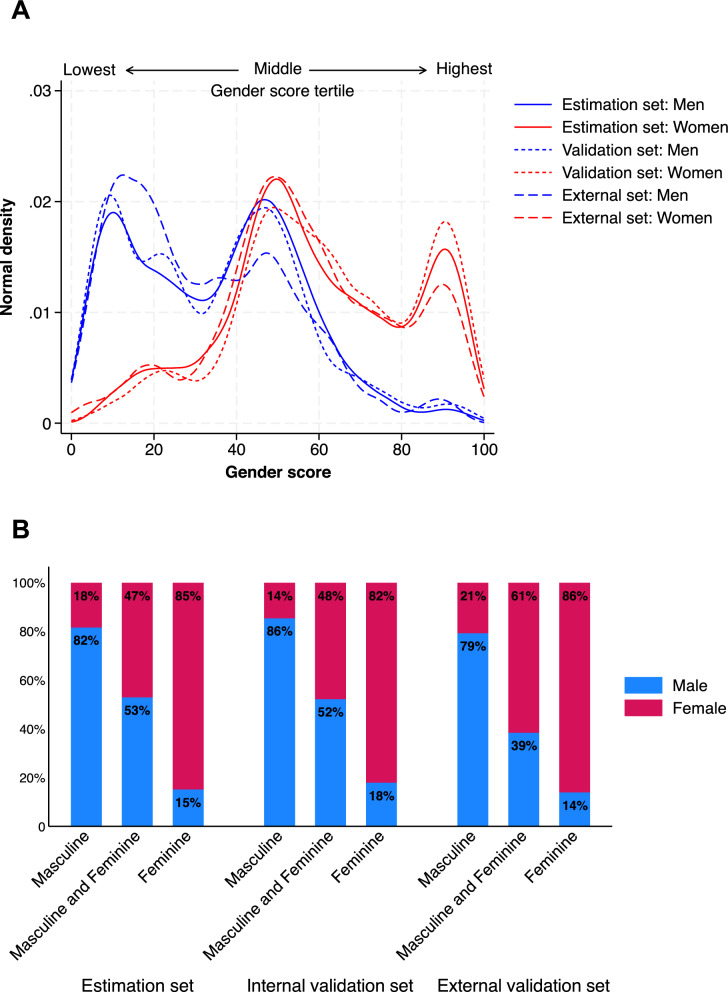
Graphs depicting the distribution of the gender score in all three datasets. Gender score as continuos variable (**A**). Datasets split into tertiles based on the gender score (**B**)

We analyzed the distribution of the composite gender score among males and females across datasets. Figure 1A shows the distribution of the gender score as a continuous variable across the estimation, validation, and external datasets. The gender score ranges from 0 (traits historically labelled as masculine) to 100 (traits historically labelled as feminine). Men predominantly clustered at the lower end of the score spectrum, whereas women were more concentrated at the higher end, with a balanced distribution observed in the middle range, indicating individuals with a mix of traits traditionally labelled as masculine and feminine. Figure 1B illustrates the distribution of the gender score across datasets, stratified into tertiles. In the first tertile, males were predominant (79–86%), while females accounted for only 14–21%. In the middle tertile, the distribution was more balanced, with males comprising 39–53% and females 47–61%. In the third tertile, females were the majority (82–86%), with only 14–18% males. This pattern was consistent across all cohorts.

To further examine the separation of gender scores between sexes across datasets, we compared the mean gender scores of males and females in each cohort (Supplementary Table 4; Supplementary Fig. 3). The mean difference between females and males was − 23.8 (95% CI − 25.7 to − 21.8) in the estimation cohort, − 27.2 (− 30.2 to − 24.2) in the internal validation cohort, and − 24.4 (− 29.0 to − 19.8) in the external validation cohort, indicating comparable separation between sexes across all three datasets.

### Predicting biological sex by sociocultural factors

**Fig. 2:**
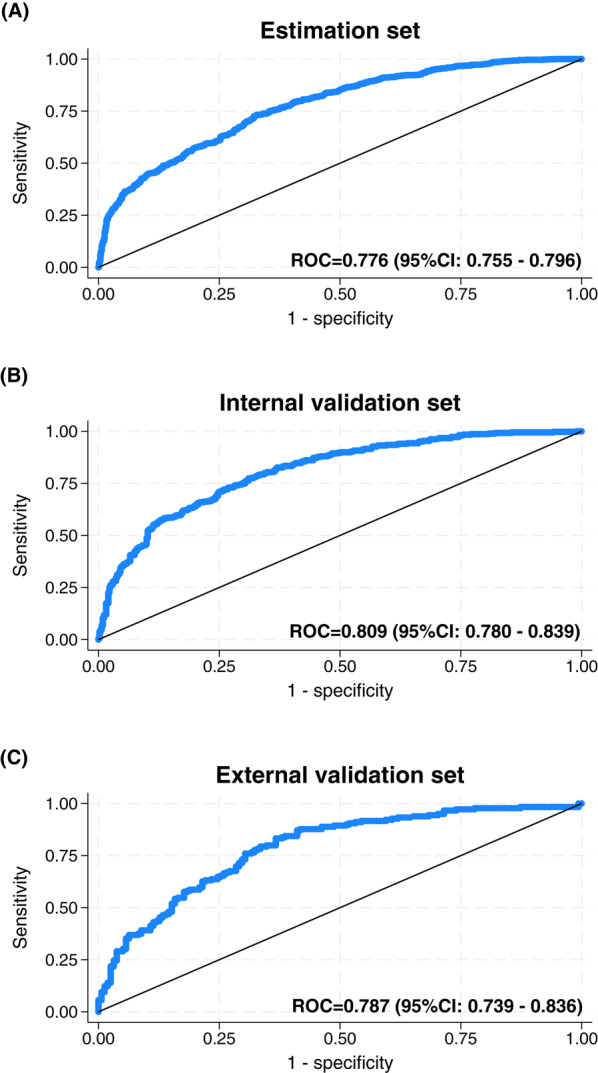
Receiver Operating Characteristic (ROC) curves from the reduced logistic regression model. Illustration shows the association between the most influential gender-related factors and biological sex. The ROC curves are shown for the estimation set (**A**), internal validation set (**B**), and external validation set (**C**)

We evaluated the ability of selected gender-related variables to predict biological sex using logistic regression models. Based on key factors identified through PCA, the full model had a ROC curve of 0.780 (Supplementary Table 5, which provides regression coefficients and ROC statistics for both the full and reduced models). A refined model, retaining only the most significant predictors, yielded a similar ROC of 0.776 (right panel, Supplementary Table 5). The final model included income (p < 0.001), household responsibility (p < 0.001), domestic stress (p < 0.001), and four BEM items. Figure 2A-C presents the ROC curves demonstrating the association between these gender-related variables and biological sex across different datasets.

### Association between sex, gender score, and prevalence of cardiometabolic and chronic health conditions

We examined the independent associations of biological sex and Gender Score tertiles (with the middle tertile as reference) with the prevalence of cardiometabolic and chronic health conditions using logistic regression models. First, age-adjusted models were constructed separately for sex and gender tertiles (Table 2). Next, fully adjusted models including age, sex, and gender tertiles were used to assess their independent contributions (Table 3). In the age-adjusted analysis, female sex was negatively associated with diabetes, hypertension, obesity, dyslipidemia, CAD, stroke, and infectious diseases, whereas no significant associations were observed for the highest (feminine) gender tertile for these conditions. Immune system disorders were positively associated with both female sex and the feminine gender tertile. Psychiatric conditions, lung diseases, and bone diseases showed positive associations with female sex but not with gender. Female sex alone accounted for 19–30% of the variance in key cardiometabolic conditions.

**Table 2:** Age-adjusted associations of sex, gender score, and gender tertiles with the prevalence of cardiometabolic and chronic health conditions

	**Female sex**			**Gender Score** **(0 = masculine-labelled traits; 100 = feminine-labelled traits)**			**Gender Score tertile** (reference: middle tertile)				
							**Lowest tertile** (more masculine-labelled traits)		**Highest tertile** (more feminine-labelled traits)		**R**^**2**^
	**OR [95% CI]**	**p-value**	**R**^**2**^	**OR [95% CI]**	**p-value**	**R**^**2**^	**OR [95% CI]**	**p-value**	**OR [95% CI]**	**p-value**	
Diabetes	0.38 [0.25, 0.56]	< 0.001	0.186	0.98 [0.92, 1.05]	0.657	0.165	1.14 [0.78, 1.67]	0.504	1.02 [0.63, 1.64]	0.935	0.165
Hypertension	0.56 [0.44, 0.72]	< 0.001	0.242	0.94 [0.90, 0.99]	0.016	0.236	1.23 [0.95, 1.60]	0.122	0.74 [0.53, 1.04]	0.083	0.237
Dyslipidemia	0.50 [0.37, 0.68]	< 0.001	0.238	0.90 [0.85, 0.95]	< 0.001	0.235	1.63 [1.20, 2.21]	0.002	0.73 [0.47, 1.12]	0.149	0.237
Obesity	0.74 [0.59, 0.93]	0.010	0.043	0.98 [0.94, 1.02]	0.376	0.040	1.14 [0.89, 1.46]	0.300	1.02 [0.76, 1.37]	0.908	0.041
Known coronary artery disease	0.18 [0.09, 0.35]	< 0.001	0.301	0.83 [0.76, 0.92]	< 0.001	0.274	2.26 [1.39, 3.70]	0.001	0.82 [0.38, 1.76]	0.614	0.274
Previous stroke/TIA	0.19 [0.06, 0.54]	0.002	0.252	0.90 [0.78, 1.04]	0.149	0.221	1.16 [0.57, 2.40]	0.678	0.61 [0.20, 1.89]	0.392	0.219
Immune system disorders (e.g., autoimmune diseases)	2.38 [1.77, 3.19]	< 0.001	0.112	1.11 [1.05, 1.17]	< 0.001	0.098	0.69 [0.49, 0.98]	0.037	1.42 [1.01, 2.01]	0.046	0.098
Psychiatric diseases	1.81 [1.08, 3.04]	0.025	0.011	1.07 [0.97, 1.18]	0.199	0.005	0.79 [0.42, 1.48]	0.456	1.39 [0.76, 2.56]	0.285	0.006
Chronic pain disorders	2.11 [0.70, 6.35]	0.183	0.013	1.10 [0.89, 1.37]	0.363	0.007	0.42 [0.09, 2.01]	0.277	1.23 [0.37, 4.10]	0.742	0.013
Pulmonary diseases (e.g., asthma, COPD, pulmonary hypertension)	1.35 [1.03, 1.77]	0.028	0.017	1.02 [0.97, 1.07]	0.509	0.015	0.89 [0.66, 1.22]	0.482	1.01 [0.72, 1.43]	0.943	0.015
Bone diseases (e.g., osteoporosis)	3.39 [1.86, 6.15]	< 0.001	0.196	1.20 [1.08, 1.34]	0.001	0.185	0.29 [0.13, 0.64]	0.002	1.24 [0.65, 2.40]	0.514	0.191
Infectious diseases (e.g., HIV, hepatitis)	0.68 [0.40, 1.15]	0.149	0.008	0.87 [0.78, 0.96]	0.009	0.016	1.14 [0.66, 1.96]	0.645	0.43 [0.18, 1.04]	0.062	0.013
Gastrointestinal diseases	1.34 [0.64, 2.79]	0.440	0.004	1.08 [0.93, 1.25]	0.314	0.006	0.81 [0.32, 2.06]	0.662	1.64 [0.69, 3.85]	0.260	0.009

Logistic regression models adjusted for age were used to estimate the associations between biological sex, gender score (as a continuous variable, change by 10 units), and gender tertiles (middle tertile as reference) with the prevalence of cardiometabolic and chronic health conditions in the overall cohort. Odds ratios (ORs) and 95% confidence intervals (CIs) are shown. TIA, transient ischemic attack; COPD, chronic obstructive pulmonary disease; HIV, human immunodeficiency virus

**Table 3:** Fully adjusted associations of sex and gender tertiles with the prevalence of cardiometabolic and chronic health conditions

	**Female sex**		**Gender Score tertile** (reference: middle tertile)				**R**^**2**^
			**Lowest tertile** (more masculine-labelled traits)		**Highest tertile** (more feminine-labelled traits)		
	**OR [95% CI]**	**p-value**	**OR [95% CI]**	**p-value**	**OR [95% CI]**	**p-value**	
Diabetes	0.27 [0.17, 0.44]	< 0.001	0.77 [0.51, 1.15]	0.195	1.73 [1.02, 2.93]	0.043	0.192
Hypertension	0.58 [0.43, 0.79]	< 0.001	1.03 [0.77, 1.36]	0.863	0.92 [0.64, 1.31]	0.631	0.243
Dyslipidemia	0.61 [0.43, 0.88]	0.008	1.37 [0.99, 1.91]	0.059	0.87 [0.55, 1.38]	0.563	0.241
Obesity	0.71 [0.55, 0.93]	0.012	1.03 [0.79, 1.34]	0.837	1.16 [0.85, 1.59]	0.352	0.044
Known coronary artery disease	0.19 [0.09, 0.39]	< 0.001	1.39 [0.83, 2.32]	0.214	1.49 [0.65, 3.43]	0.344	0.303
Previous stroke/TIA	0.15 [0.05, 0.48]	0.001	0.70 [0.33, 1.47]	0.345	1.13 [0.34, 3.77]	0.844	0.255
Immune system disorders (e.g., autoimmune diseases)	2.26 [1.60, 3.19]	< 0.001	0.95 [0.66, 1.39]	0.807	1.09 [0.76, 1.56]	0.650	0.112
Psychiatric diseases	1.67 [0.92, 3.03]	0.089	0.93 [0.48, 1.82]	0.834	1.17 [0.62, 2.21]	0.630	0.011
Chronic pain disorders	1.73 [0.52, 5.76]	0.371	0.50 [0.10, 2.48]	0.394	1.02 [0.29, 3.60]	0.974	0.018
Pulmonary diseases (e.g., asthma, COPD, pulmonary hypertension)	1.40 [1.03, 1.90]	0.032	1.00 [0.72, 1.39]	0.998	0.90 [0.63, 1.29]	0.563	0.017
Bone diseases (e.g., osteoporosis)	2.40 [1.21, 4.76]	0.013	0.43 [0.18, 1.03]	0.058	0.97 [0.49, 1.90]	0.920	0.204
Infectious diseases (e.g., HIV, hepatitis)	0.85 [0.48, 1.53]	0.599	1.08 [0.61, 1.92]	0.780	0.46 [0.19, 1.14]	0.094	0.014
Gastrointestinal diseases	1.05 [0.45, 2.46]	0.916	0.82 [0.31, 2.17]	0.695	1.61 [0.65, 4.01]	0.307	0.009

Logistic regression models adjusted for age, biological sex, and gender tertiles (middle tertile as reference) were used to assess their independent associations with the prevalence of cardiometabolic and chronic health conditions. Odds ratios (ORs) and 95% confidence intervals (CIs) are presented. TIA, transient ischemic attack; COPD, chronic obstructive pulmonary disease; HIV, human immunodeficiency virus

In the fully adjusted models including both sex and gender, divergent associations emerged for diabetes: female sex remained negatively associated, while feminine gender showed a positive association. Female sex remained significantly and negatively associated with hypertension, dyslipidemia, obesity, CAD, and stroke, independent of gender. Conversely, immune system disorders, lung diseases, and bone diseases retained their positive association with female sex, with no significant contribution from gender.

### Associations between biological sex and health conditions after matching for gender

To isolate the effect of biological sex, we compared associations with cardiometabolic and chronic health conditions in males and females matched for gender score. Specifically, we applied propensity score matching, pairing females and males with identical gender scores (1:1 matching, n = 2,446), and conducted a conditional logistic regression analysis (Table 4**).** The mean gender score in the matched cohort was 50.3 ± 22.5, indicating that most matched individuals were located in the middle tertile of the gender score distribution. After controlling for gender, female sex remained negatively associated with diabetes, hypertension, dyslipidemia, obesity, CAD, and stroke, while it was positively associated with immune system disorders, psychiatric diseases, pulmonary diseases, and bone diseases. Notably, the negative association between female sex and diabetes weakened after gender matching, indicating opposing effects of sex and gender on diabetes risk.

**Table 4:** Conditional logistic regression models showing the association of sex with clinical outcomes after propensity score matching pairing females and males with identical gender scores (1:1 matching, n = 2,446)

	**OR [95% CI]**	**p-value**
Diabetes	0.36 [0.24, 0.54]	< 0.001
Hypertension	0.56 [0.43, 0.73]	< 0.001
Dyslipidemia	0.52 [0.37, 0.72]	< 0.001
Obesity	0.77 [0.61, 0.98]	0.031
Known coronary artery disease	0.15 [0.05, 0.41]	< 0.001
Previous stroke/TIA	0.20 [0.05, 0.81]	0.024
Immune system disorders (e.g., autoimmune diseases)	2.18 [1.61, 2.97]	< 0.001
Psychiatric diseases	1.78 [1.03, 3.07]	0.038
Chronic pain disorders	1.79 [0.60, 5.36]	0.300
Pulmonary diseases (e.g., asthma, COPD, pulmonary hypertension)	1.45 [1.08, 1.94]	0.012
Bone diseases (e.g., osteoporosis)	3.29 [1.72, 6.28]	< 0.001
Infectious diseases (e.g., HIV, hepatitis)	0.76 [0.42, 1.37]	0.361
Gastrointestinal diseases	1.40 [0.64, 3.07]	0.398

TIA, transient ischemic attack; COPD, chronic obstructive pulmonary disease; HIV, human immunodeficiency virus

### Associations of individual gender-related traits with the prevalence of cardiometabolic and health conditions

We explored how specific gender-related traits contributed to the risk of cardiometabolic and health conditions compared to the composite Gender Score. To assess whether gender is better captured as a summary score or through individual items, we repeated the logistic regression analysis using single gender-related variables instead of the composite score (Supplementary Table 6A-B**,** which detail the associations of each item with both cardiometabolic [6A] and non-cardiometabolic health conditions [6B]). Domestic stress was positively associated with diabetes, obesity, and psychiatric diseases. In contrast, the BEM trait “being independent” was associated to a lower risk of diabetes, dyslipidemia, obesity, immune system disorders, and bone disease, while risk-taking behavior was linked to reduced risk of diabetes and hypertension. Among the four selected key sociocultural variables, at least one variable was associated with the health conditions investigated in our study. However, none of the individual items showed a stronger correlation with biological sex than the composed Gender Score. These findings suggest that while biological sex broadly influences the risk of cardiometabolic and health conditions, individual gender-related traits uniquely contribute selectively to specific conditions. Developing a composite Gender Score may therefore provide a more robust approach to characterizing gender-related influences on health. To explore potential sex-specific effects of individual gender-related variables, we additionally performed sex-stratified analyses for the models presented in Supplementary Tables 6A and 6B (Supplementary Tables 7A and 7B). Overall, the direction of associations was largely consistent with the combined analyses, although several associations lost statistical significance due to reduced statistical power within sex strata. No individual gender-related variable showed a consistent or qualitatively different association between women and men. These findings support the robustness of the main analyses and indicate that the observed associations were not driven by sex-specific effects of individual gender-related variables.

## Discussion

In this Swiss cohort, women and men differed across several sociocultural characteristics, enabling the construction of a composite Gender Score comparable to those previously developed in Canadian and German populations. This score allowed us to examine sex and gender as related but distinct dimensions within regression models. Across age-adjusted and fully adjusted analyses, biological sex showed a consistently stronger association than gender with most cardiometabolic and health conditions, including hypertension, dyslipidemia, CAD, stroke, immune disorders, psychiatric conditions, and bone diseases. In contrast, gender demonstrated more limited and outcome-specific associations. One condition in which sex and gender showed opposing associations was diabetes. Analyses matching women and men on gender score further reinforced the dominant contribution of biological sex to the overall risk of cardiometabolic and health conditions, while illustrating that sex and gender may exert divergent effects for selected conditions.

By combining key sociocultural variables identified through PCA, the Gender Score accurately predicted biological sex within our Swiss cohort. This highlights the relevance of these variables in distinguishing men and women in contemporary Swiss society. Our approach conceptualizes gender as a continuum of sociocultural traits historically labelled as masculine and feminine, allowing varying combinations of these traits to be represented across individuals, including cisgender and transgender persons. However, this framework is not designed to capture identities such as gender-fluid or agender, which fall outside a binary or spectrum-based model. Our gender framework and questionnaire are based on the concept of gender-related traits historically labelled as masculine and feminine in relation to biomedical disease, as originally proposed by Pelletier et al. [5]. This approach captures a spectrum of gender-related traits between these two poles but does not encompass non-gendered or gender-fluid identities. Despite this limitation—and the ongoing lack of a standardized method for measuring gender [2, 5–7, 11, 17–22] — our 7-item gender score effectively classified the vast majority of individuals in our cohort as biological males or females and revealed meaningful associations with common health conditions.

The distribution of the Gender Score in our cohort was comparable to that reported in an elderly German population, showing a broad range of Gender Scores [12]. Notably, biological females were more likely to exhibit lower Gender Scores than biological males were to exhibit higher scores, a pattern consistent with previous observations [20]. This asymmetry may partly reflect the age-dependent and context-specific nature of gender constructs. In the younger age group (< 45 years), both women and men in the external validation cohort more frequently exhibited lower Gender Scores, reflecting traits traditionally labelled as masculine, compared with older individuals. Overall, a larger proportion of participants in our cohort fell into the middle gender score tertile than in older cohorts [12], reflecting a greater overlap of traits traditionally labelled as masculine and feminine. This distribution likely attenuated associations between gender and cardiometabolic and health conditions and motivated our additional analyses using gender tertiles.

We found that female biological sex was strongly negatively associated with diabetes, hypertension, obesity, dyslipidemia, CAD, and stroke, and positively associated with lung diseases, immune system disorders, psychiatric conditions, and bone diseases. To confirm the validity of these associations, we applied a second statistical approach—propensity score matching—pairing males and females with identical gender scores. This method removes gender as a confounding factor, attributing any remaining differences to biological sex. Although our results do not imply causal relationships, the reanalysis confirmed these associations, underscoring the importance of including biological sex as a critical variable in clinical studies [23].

One observation in our study was the divergent association of sex and gender with diabetes risk. This result should be interpreted cautiously, as diabetes was one of the few outcomes showing a statistically significant gender effect and may partly reflect chance variation in a cross-sectional analysis. While female sex was associated with a lower likelihood of diabetes, a more feminine gender profile—characterized by sociocultural traits such as higher domestic stress and caregiving responsibilities—was independently linked to increased diabetes risk. This pattern is consistent with findings by Pohrt et al., who reported associations between feminine gender characteristics and metabolic conditions and obesity, and aligns with the emerging hypothesis that gendered exposures may attenuate or even counteract the biological advantage typically conferred by female sex in metabolic health [12, 24]. Nonetheless, given the cross-sectional design of our study, these results reflect associations rather than causal relationships.

Beyond diabetes, gender showed limited independent associations with the prevalence of cardiometabolic and health conditions in this cohort, whereas biological sex consistently explained a substantial proportion of variance across multiple cardiometabolic conditions. This pattern suggests that, in this Swiss population, biological sex may currently outweigh gender-related factors in shaping the distribution of cardiometabolic and chronic health conditions. It also raises the possibility that in comparatively less gender-polarized societies, the health impact of gender—as captured by traditional sociocultural measures—may be attenuated. Alternatively, relevant gender-related influences may operate through dimensions not captured in our study, such as workplace structures, healthcare access, discrimination, or life-course exposures, which warrant further investigation. These observations may therefore reflect both the sociocultural context of this population and the specific gender indicators used in our analysis.

Our additional analyses using gender tertiles further highlight the importance of disaggregating sex and gender. Opposing associations with diabetes became more apparent in fully adjusted models, whereas previously observed associations of female sex with psychiatric disease and of feminine gender with immune disorders were no longer significant. These shifts suggest overlapping contributions of sex and gender and potential confounding in simpler models, underscoring the need for multivariable approaches when examining their independent effects.

We also examined whether individual gender-related variables provided stronger associations with cardiometabolic and chronic health conditions than the composite Gender Score. While traits such as domestic stress, income, household responsibilities, and the BEM trait “independence” were associated with several conditions, these associations were not always intuitive. Some traits traditionally considered detrimental appeared protective, likely reflecting residual confounding by unmeasured factors such as environmental influences, health behaviors, or access to care. These analyses of individual gender-related variables should therefore be considered exploratory and interpreted with caution. Importantly, no single item outperformed the composite Gender Score or biological sex in predicting cardiometabolic and chronic health conditions, suggesting that integrating multiple sociocultural traits enhances robustness and interpretability. Developing and validating tools to assess gender-related traits in prospective clinical studies may help refine risk assessment and inform future therapeutic strategies. Overall, our findings support the relevance of integrated sex–gender frameworks for understanding cardiometabolic risk.

Our study has several limitations. By design, it included patients with COVID-19 and their comorbidities at a single time point, limiting our ability to assess disease progression or treatment effects. In addition, reverse causation cannot be excluded; for example, chronic conditions such as diabetes may influence gender-related exposures captured by the Gender Score, including stress levels or social roles. Even after adjustment for age, sex and Gender Score explained up to 30% of the variance in comorbidity prevalence, indicating that additional, unmeasured factors likely contribute to clinical outcomes. We anticipate that gender-related effects may be even more pronounced when considering longitudinal outcomes and treatment responses, which warrant further investigation. Only 45% of the original cohort had complete data available for the present analysis, which may introduce selection bias and limit external validity. Participants included in the analysis therefore represent a selected subset of the original cohort. In addition, the study population consisted of individuals with confirmed COVID-19 infection, which may further limit generalizability to broader populations. While this is unlikely to affect internal validity, the external validity of the findings should be interpreted with caution.

The assessment of gender remains challenging, as no universally accepted methodology exists. Our study was constrained by the number of questions that could be asked to ill patients, precluding the use of more extensive gender instruments. The Gender Score is based on sociocultural variables historically associated with masculinity and femininity, derived from the BEM and Pilote gender frameworks, which conceptualize gender along a continuum within a given sociocultural context [5, 15]. As such, the score may not fully capture contemporary gender diversity, including non-binary or gender-fluid identities. Importantly, the score primarily reflects gendered social roles and traits rather than gender identity. Although we use terms such as “masculine” and “feminine” traits, these refer to culturally shaped role characteristics reported by individuals rather than inherent qualities tied to biological sex. Future studies should aim to incorporate measures of gender identity alongside gendered social roles and norms to better capture the full complexity of gender. Furthermore, associations were limited to cardiometabolic and infectious conditions, and generalizability to other health conditions remains to be established. As the Gender Score reflects sociocultural norms in Switzerland and relies partly on self-reported social roles and traits, reproducibility across settings may be influenced by recall or social desirability bias. In addition, gender-related sociocultural indicators vary across societies; therefore, Gender Scores developed in one population should be contextually validated and potentially recalibrated before application in other cultural settings.

A key strength of our study is its large, well-characterized Swiss cohort, designed as a prospective study with a systematic approach to analyzing both sex and gender-related factors.

In summary, our findings show that biological sex and sociocultural gender contribute differently to cardiometabolic and chronic health conditions, with biological sex explaining most associations in this cohort and gender exerting more limited, condition-specific effects. For example, diabetes showed opposing associations for sex and gender, illustrating that these constructs are related but not interchangeable. Overall, our results emphasize the value of sex–gender frameworks for improving epidemiological interpretation, while underscoring the need for longitudinal studies and more inclusive gender measures to clarify when sociocultural factors meaningfully enhance risk assessment beyond biological sex.

## Supplementary Information

Additional file 1.

## Data Availability

Based on the Business Administration System for Ethics Committees (BASEC) ethics approval, the non-anonymized raw data cannot be shared publicly. However, anonymised data that underlie the results reported in this article will become available to interested parties for non-commercial reasons, after the publication upon reasonable requests made to the corresponding author. Data requestors will need to sign a data access agreement.

## Abbreviations

AIC: Akaike Information Criterion
BIC: Bayesian Information Criterion
CAD: Coronary artery disease
CI: Confidence Interval
COVID-19: Coronavirus Disease 2019
CVD: Cardiovascular Disease
MP: Multiprocessor
OR: Odds ratios
PCA: Principal component analysis
SARS-CoV-2: Severe Acute Respiratory Syndrome Coronavirus 2
