# Supplementary material for: Decoding sex and gender effects on health: evidence from a nationwide cohort
Source: Biol Sex Differ. 2026 Mar 30;17:100. doi: 10.1186/s13293-026-00888-8 (PMC13154686; doi:10.1186/s13293-026-00888-8)
Supplement: Supplementary file 1 — Additional file 1. [file 13293_2026_888_MOESM1_ESM.docx]

**Supplementary Material**

**Content**

1. **Supplementary Tables**

- **Supplementary Table 1:** Socioeconomic characteristics of the estimation, internal validation, and external validation cohort stratified by sex.
  - **Supplementary Table 2:** Correlation between gender-related variables within the estimation cohort.
  - **Supplementary Table 3:** Principal component analysis.
  - **Supplementary Table 4:** Mean and median gender scores by sex across cohorts
  - **Supplementary Table 5:** Logistic regression coefficients of gender-related factors associated with biological sex performed in the estimation set.
  - **Supplementary Table 6:** Association between biological sex and individual items from the gender score with cardiometabolic and health conditions
  - **Supplementary Table 7:** Association between individual items from the gender score with cardiometabolic and health conditions stratified by sex

1. **Supplementary Figures**
   - **Supplementary Figure 1:** Gender-related questions included in the questionnaire.
   - **Supplementary Figure 2:** Flowchart providing an overview of the estimation and validation data sets.
   - **Supplementary Figure 3:** Violin plots of gender score distribution by sex across datasets

**Supplementary Table 1: Socioeconomic characteristics of the estimation, internal validation, and external validation cohort stratified by sex.** SD, standard deviation; BEM score, measure used to assess gender roles.

| **Gender-Related Variables** | **Estimation set (n = 1,882)** | | | **Internal validation set (n = 808)** | | | **External validation set (n = 337)** | | |
| --- | --- | --- | --- | --- | --- | --- | --- | --- | --- |
|  | **Male**  **(n = 1,026)** | **Female**  **(n = 856)** | **p-value** | **Male**  **(n = 441)** | **Female**  **(n = 367)** | **p-value** | **Male**  **(n = 158)** | **Female**  **(n = 179)** | **p-value** |
| Education |  |  | 0.59 |  |  | 0.37 |  |  | 0.22 |
| No education qualification (%) | 61 (5.9) | 50 (5.8) |  | 21 (4.8) | 18 (4.9) |  | 5 (3.2) | 6 (3.4) |  |
| Primary education (%) | 55 (5.4) | 59 (6.9) |  | 25 (5.7) | 31 (8.4) |  | 11 (7.0) | 15 (8.4) |  |
| Secondary education or vocational degree (%) | 403 (39.3) | 332 (38.8) |  | 174 (39.5) | 150 (40.9) |  | 80 (50.6) | 107 (59.8) |  |
| University or technical college degree (%) | 507 (49.4) | 415 (48.5) |  | 221 (50.1) | 168 (45.8) |  | 62 (39.2) | 51 (28.5) |  |
| Marital status |  |  | 0.52 |  |  | 0.028 |  |  | 0.067 |
| married/partnership (%) | 700 (68.2) | 572 (66.8) |  | 320 (72.6) | 240 (65.4) |  | 121 (76.6) | 121 (67.6) |  |
| living alone (%) | 326 (31.8) | 284 (33.2) |  | 121 (27.4) | 127 (34.6) |  | 37 (23.4) | 58 (32.4) |  |
| Income |  |  | <0.001 |  |  | <0.001 |  |  | <0.001 |
| Earns highest income in household (%) | 717 (69.9) | 365 (42.6) |  | 300 (68.0) | 138 (37.6) |  | 109 (69.0) | 85 (47.5) |  |
| Earns lowest income in household (%) | 166 (16.2) | 349 (40.8) |  | 72 (16.3) | 167 (45.5) |  | 17 (10.8) | 63 (35.2) |  |
| Equal between partners (%) | 143 (13.9) | 142 (16.6) |  | 69 (15.6) | 62 (16.9) |  | 32 (20.3) | 31 (17.3) |  |
| Main person responsible for household work |  |  | <0.001 |  |  | <0.001 |  |  | <0.001 |
| No (%) | 334 (32.6) | 108 (12.6) |  | 151 (34.2) | 47 (12.8) |  | 43 (27.2) | 18 (10.1) |  |
| Yes (%) | 318 (31.0) | 481 (56.2) |  | 125 (28.3) | 207 (56.4) |  | 45 (28.5) | 113 (63.1) |  |
| Equal distribution between partners (%) | 374 (36.5) | 267 (31.2) |  | 165 (37.4) | 113 (30.8) |  | 70 (44.3) | 48 (26.8) |  |
| Average domestic stress level (score 0 - 10) – Mean (SD) | 3.0 (2.0) | 3.7 (2.3) | <0.001 | 3.0 (1.8) | 3.9 (2.5) | <0.001 | 2.8 (1.8) | 3.3 (2.2) | 0.034 |
| Main responsibility for childcare/care of family members (min 1 - max 6) – mean (SD) | 1.7 (2.1) | 1.8 (2.3) | 0.13 | 1.6 (2.1) | 2.1 (2.3) | 0.005 | 1.5 (1.9) | 1.7 (2.1) | 0.56 |
| BEM score (overall) | 5.0 (0.9) | 4.9 (0.9) | 0.006 | 5.0 (1.0) | 4.9 (0.9) | 0.19 | 4.9 (0.8) | 4.9 (0.9) | 0.65 |
| I am someone who …  (min 1 – max 7) – mean (SD) |  |  |  |  |  |  |  |  |  |
| …defends his own opinion. | 5.4 (1.3) | 5.5 (1.4) | 0.39 | 5.5 (1.4) | 5.5 (1.4) | 0.87 | 5.3 (1.3) | 5.5 (1.3) | 0.30 |
| …has leadership qualities. | 5.2 (1.4) | 4.8 (1.6) | <0.001 | 5.1 (1.5) | 4.8 (1.5) | 0.004 | 5.1 (1.2) | 4.8 (1.5) | 0.026 |
| …is independent. | 5.6 (1.4) | 5.7 (1.4) | 0.12 | 5.6 (1.4) | 5.7 (1.4) | 0.22 | 5.5 (1.4) | 5.7 (1.3) | 0.18 |
| …is willing to take risks. | 4.6 (1.6) | 4.3 (1.6) | <0.001 | 4.6 (1.6) | 4.2 (1.6) | 0.002 | 4.7 (1.6) | 4.5 (1.4) | 0.31 |
| …is positive. | 5.5 (1.4) | 5.5 (1.4) | 0.96 | 5.4 (1.4) | 5.6 (1.4) | 0.073 | 4.5 (1.4) | 4.8 (1.7) | 0.020 |
| …is assertive. | 5.3 (1.3) | 5.2 (1.3) | 0.19 | 5.2 (1.4) | 5.2 (1.3) | 0.76 | 5.3 (1.2) | 5.3 (1.2) | 0.56 |
| …has a strong personality. | 5.4 (1.3) | 5.4 (1.4) | 0.64 | 5.4 (1.4) | 5.4 (1.3) | 0.63 | 5.2 (1.2) | 5.4 (1.2) | 0.19 |
| …is ready to take a stand. | 5.5 (1.3) | 5.3 (1.4) | 0.012 | 5.5 (1.3) | 5.4 (1.3) | 0.39 | 5.5 (1.2) | 5.6 (1.2) | 0.31 |
| …is energetic. | 4.8 (1.5) | 4.8 (1.6) | 0.74 | 4.8 (1.5) | 4.7 (1.5) | 0.29 | 4.8 (1.4) | 4.9 (1.4) | 0.35 |
| …is aggressive. | 2.6 (1.4) | 2.3 (1.4) | <0.001 | 2.6 (1.5) | 2.3 (1.4) | 0.001 | 2.8 (1.5) | 2.5 (1.4) | 0.040 |
| Self-assessment of gender identity (scale 1–7; 1 = predominantly traits traditionally labelled as masculine, 7 = predominantly traits traditionally labelled as feminine), mean (SD) | 2.4 (1.4) | 5.5 (1.3) | <0.001 | 2.3 (1.4) | 5.5 (1.3) | <0.001 | 2.1 (1.1) | 5.5 (1.6) | <0.001 |

**Supplementary Table 2: Correlation between gender-related variables within the estimation cohort (n=1,882).** Correlation coefficient for each variable is presented. BEM score, measure used to assess gender roles.

| (n = 1,882) | Education | Marital status | Income | Main person responsible for household work | Average domestic stress level | Main responsibility for childcare/care of family members | …defends his own opinion. | …has leadership qualities | …is independent | …is willing to take risks | …is positive. | …is assertive. | …has a strong personality. | …is ready to take a stand. | …is energetic. |
| --- | --- | --- | --- | --- | --- | --- | --- | --- | --- | --- | --- | --- | --- | --- | --- |
| Education | 1 |  |  |  |  |  |  |  |  |  |  |  |  |  |  |
| Marital status | -0.0122 | 1 |  |  |  |  |  |  |  |  |  |  |  |  |  |
| Income | -0.0500 | -0.2881 | 1 |  |  |  |  |  |  |  |  |  |  |  |  |
| Main person responsible for household work | 0.1167 | -0.2214 | 0.2374 | 1 |  |  |  |  |  |  |  |  |  |  |  |
| Average domestic stress level | -0.0615 | -0.0758 | 0.0535 | 0.0097 | 1 |  |  |  |  |  |  |  |  |  |  |
| Main responsibility for childcare/care of family members | -0.1041 | -0.2502 | 0.0634 | 0.0258 | 0.3234 | 1 |  |  |  |  |  |  |  |  |  |
| Personal characteristics according to BEM score.  I am someone who… |  |  |  |  |  |  |  |  |  |  |  |  |  |  |  |
| …defends his own opinion. | 0.0624 | -0.0427 | -0.0029 | 0.0142 | -0.0434 | 0.0439 | 1 |  |  |  |  |  |  |  |  |
| …has leadership qualities. | 0.1683 | -0.0728 | -0.0875 | 0.0621 | -0.0493 | 0.0334 | 0.4618 | 1 |  |  |  |  |  |  |  |
| …is independent. | 0.1876 | 0.0573 | -0.0918 | 0.0624 | -0.1226 | -0.0347 | 0.4235 | 0.3781 | 1 |  |  |  |  |  |  |
| …is willing to take risks. | 0.1132 | 0.0457 | -0.0725 | -0.0137 | -0.0353 | -0.0208 | 0.2908 | 0.3489 | 0.3883 | 1 |  |  |  |  |  |
| …is positive. | 0.0659 | -0.0300 | -0.0299 | 0.0599 | -0.1540 | 0.0568 | 0.3016 | 0.3147 | 0.3767 | 0.3459 | 1 |  |  |  |  |
| …is assertive. | 0.0462 | -0.0223 | -0.0566 | 0.0189 | -0.0785 | 0.0371 | 0.5289 | 0.5193 | 0.4147 | 0.3755 | 0.4460 | 1 |  |  |  |
| …has a strong personality. | 0.0523 | -0.0349 | -0.0214 | 0.0453 | -0.0435 | 0.0535 | 0.5267 | 0.5451 | 0.4549 | 0.3618 | 0.4553 | 0.6877 | 1 |  |  |
| …is ready to take a stand. | 0.1444 | -0.0284 | -0.0526 | 0.0280 | -0.0808 | 0.0457 | 0.5631 | 0.5580 | 0.4599 | 0.3756 | 0.4026 | 0.6187 | 0.6784 | 1 |  |
| …is energetic. | 0.0491 | -0.0110 | -0.0540 | 0.0028 | 0.0566 | 0.0878 | 0.4008 | 0.3823 | 0.3044 | 0.3672 | 0.3026 | 0.4403 | 0.4996 | 0.4909 | 1 |
| …is aggressive. | -0.0031 | -0.0393 | -0.0240 | -0.0171 | 0.1477 | 0.0707 | 0.0661 | 0.0928 | -0.0841 | 0.1381 | -0.1369 | 0.0592 | 0.0787 | 0.0684 | 0.2688 |

**Supplementary Table 3: Principal component analysis (PCA).** PCA performed on the estimation cohort (n=1,882), with factor loadings for ten principal components (PCs) presented. The PCs are ranked according to the variance they capture. A total of 13 gender-related variables were found to load onto the 10 retained PCs, with the identified factors highlighted in bold.

| **Gender-related factors** | **Principal Components (PCs)** | | | | | | | | | |
| --- | --- | --- | --- | --- | --- | --- | --- | --- | --- | --- |
|  | **1** | **2** | **3** | **4** | **5** | **6** | **7** | **8** | **9** | **10** |
| Education | -0.0049 | 0.0043 | 0.0096 | -0.0179 | 0.0081 | -0.0058 | **0.9824** | 0.0063 | -0.0087 | -0.0113 |
| Marital status | 0.0072 | -0.0065 | 0.0239 | -0.0489 | 0.0817 | **0.8763** | -0.0063 | -0.0795 | -0.0174 | -0.1181 |
| Income | 0.0133 | -0.0132 | 0.0082 | -0.0019 | 0.0369 | -0.0574 | 0.0082 | **0.9471** | 0.0045 | -0.0528 |
| Main person responsible for household work | -0.0065 | 0.0167 | 0.0134 | -0.0257 | 0.0144 | -0.0123 | -0.0087 | 0.0035 | **0.9855** | -0.0164 |
| Domestic stress level | -0.0044 | -0.0352 | -0.0539 | 0.0511 | **0.9343** | 0.0613 | 0.0097 | 0.0366 | 0.0175 | 0.0761 |
| Main responsibility for childcare/care of family members | 0.0031 | 0.0308 | 0.0769 | -0.0836 | 0.1053 | -0.1222 | -0.0180 | -0.0718 | -0.0278 | **0.8610** |
| BEM score (overall)  I am someone who …  (min 1 – max 7) – mean (SD) |  |  |  |  |  |  |  |  |  |  |
| …defends his own opinion. | **0.5310** | -0.0166 | -0.3187 | -0.0237 | -0.0872 | 0.0438 | -0.0562 | 0.1166 | -0.0542 | 0.1189 |
| …has leadership qualities. | 0.3587 | -0.0169 | 0.0118 | 0.0263 | 0.1678 | -0.3295 | 0.0660 | -0.2533 | 0.0270 | -0.2638 |
| …is independent. | 0.3097 | -0.2259 | -0.3090 | **0.4513** | -0.1911 | 0.2112 | 0.0773 | 0.0024 | 0.1292 | 0.2844 |
| …is willing to take risks. | -0.0483 | 0.0869 | 0.1084 | **0.8606** | 0.0890 | -0.0772 | -0.0376 | -0.0036 | -0.0561 | -0.1100 |
| …is positive. | -0.0258 | -0.1574 | **0.7442** | 0.1365 | -0.1297 | 0.0467 | 0.0263 | 0.0208 | 0.0350 | 0.1347 |
| …is assertive. | 0.3626 | -0.0009 | 0.2558 | -0.0655 | 0.0562 | -0.0490 | -0.0894 | -0.0343 | -0.0204 | -0.1259 |
| …has a strong personality. | 0.3855 | 0.0313 | 0.2516 | -0.0934 | 0.0721 | 0.0088 | -0.0554 | 0.0228 | 0.0157 | -0.0768 |
| …is ready to take a stand. | **0.4196** | 0.0337 | 0.1003 | -0.0754 | 0.0002 | -0.0059 | 0.0757 | 0.0053 | -0.0349 | -0.0209 |
| …is energetic. | 0.1827 | **0.4380** | 0.2725 | 0.0112 | -0.0020 | 0.2086 | 0.0436 | 0.0962 | 0.0028 | 0.1364 |
| …is aggressive. | -0.0245 | **0.8482** | -0.1201 | 0.0633 | -0.0482 | -0.0277 | 0.0022 | -0.0305 | 0.0277 | 0.0127 |

**Supplementary Table 4. Sex-specific distribution of the composite gender score across study cohorts.** Mean (standard deviation) and median (interquartile range) values of the gender score are shown separately for males and females in the estimation, internal validation, and external validation cohorts. The mean difference between males and females with corresponding 95% confidence intervals is reported. The gender score ranges from 0 to 100, with higher values indicating a greater expression of traits historically labelled as feminine and lower values indicating traits historically labelled as masculine.

|  |  | **Male** | **Female** | **Mean difference (95%CI)** |
| --- | --- | --- | --- | --- |
| **Estimation set** | Mean (SD) | 35.3 (20.6) | 59.1 (22.3) | -23.8 (-25.7, -21.8) |
|  | Median (IQR) | 37.4 (16.6-50.4) | 56.3 (45.3-77.6) |  |
| **Internal validation set** | Mean (SD) | 34.6 (21.3) | 61.9 (22.0) | -27.2 (-30.2, -24.2) |
|  | Median (IQR) | 35.6 (14.9-49.2) | 60.0 (46.8-83.0) |  |
| **External validation set** | Mean (SD) | 32.7 (20.7) | 57.1 (21.9) | -24.4 (-29.0, -19.8) |
|  | Median (IQR) | 30.0 (15.3-48.0) | 55.5 (44.2-74.1) |  |

**Supplementary Table 5: Logistic regression coefficients of gender-related factors associated with biological sex performed in the estimation set (n = 1,882).** A full model derived from the principal component analysis( PCA) as well as a reduced model, containing only factors showing significant associations with biological sex in the full model, are presented. BEM score, measure used to assess gender roles. AIC, Akaike information criterion. BIC, Bayesian information criterion.

| **Gender-related factors** | **Full model derived from PCA** | | **Reduced model** | |
| --- | --- | --- | --- | --- |
|  | **Coefficient estimate** | **p-value** | **Coefficient estimate** | **p-value** |
| Intercept | -3.372 |  | -2.963 |  |
| Education |  |  |  |  |
| No education qualification | Reference |  |  |  |
| Primary education | 0.435 | 0.164 |  |  |
| Secondary education or vocational degree | 0.305 | 0.208 |  |  |
| University or technical college degree | 0.331 | 0.168 |  |  |
| Marital status |  |  |  |  |
| married/partnership | Reference |  |  |  |
| living alone | -0.041 | 0.759 |  |  |
| Income |  |  |  |  |
| Earns highest income in household | Reference |  | Reference |  |
| Earns lowest income in household | 2.110 | <0.001 | 2.092 | <0.001 |
| Equal between partners | 1.191 | <0.001 | 1.184 | <0.001 |
| Main person responsible for household work |  |  |  |  |
| No | Reference |  | Reference |  |
| Yes | 2.200 | <0.001 | 2.197 | <0.001 |
| Equal distribution between partners | 0.956 | <0.001 | 0.965 | <0.001 |
| Main responsibility for childcare/care of family members | -0.032 | 0.248 |  |  |
| Average domestic stress level (score 0 - 10) | 0.144 | <0.001 | 0.134 | <0.001 |
| BEM score (overall)  I am someone who …  (min 1 – max 7) – mean (SD) |  |  |  |  |
| …defends his own opinion. | 0.107 | 0.029 | 0.117 | 0.015 |
| …is independent. | 0.176 | <0.001 | 0.192 | <0.001 |
| …is willing to take risks. | -0.144 | <0.001 | -0.120 | 0.002 |
| …is positive. | 0.052 | 0.262 |  |  |
| …is ready to take a stand. | -0.149 | 0.007 | -0.109 | 0.036 |
| …is energetic. | 0.067 | 0.121 |  |  |
| …is aggressive. | -0.137 | 0.001 | -0.133 | 0.001 |
| AIC | 2,122.45 |  | 2,115.86 |  |
| BIC | 2,222.17 |  | 2,176.80 |  |
| ROC | 0.780 |  | 0.776 |  |

|  | **Diabetes** | | **Hypertension** | | **Dyslipidemia** | | **Obesity**  **(BMI > 30kg/m^2^)** | | **Known coronary artery disease** | | **Previous stroke/TIA** | |
| --- | --- | --- | --- | --- | --- | --- | --- | --- | --- | --- | --- | --- |
|  | **OR [95%CI]** | **p-value** | **OR [95%CI]** | **p-value** | **OR [95%CI]** | **p-value** | **OR [95%CI]** | **p-value** | **OR [95%CI]** | **p-value** | **OR [95%CI]** | **p-value** |
| Female sex | **0.29 [0.18, 0.45]** | **<0.001** | **0.58 [0.45, 0.75]** | **<0.001** | **0.57 [0.42, 0.79]** | **<0.001** | **0.69 [0.53, 0.89]** | **0.005** | **0.23 [0.11, 0.45]** | **<0.001** | **0.16 [0.05, 0.50]** | **0.001** |
| Income |  |  |  |  |  |  |  |  |  |  |  |  |
| Earns highest income in household | Reference |  | Reference |  | Reference |  | Reference |  | Reference |  | Reference |  |
| Earns lowest income in household | 1.10 [0.72, 1.67] | 0.660 | **0.62 [0.47, 0.84]** | **0.002** | **0.57 [0.40, 0.82]** | **0.002** | 0.82 [0.62, 1.09] | 0.175 | **0.51 [0.27, 0.96]** | **0.037** | 0.64 [0.24, 1.71] | 0.375 |
| Equal between partners | 0.83 [0.47, 1.44] | 0.502 | 0.92 [0.66, 1.29] | 0.637 | 0.90 [0.61, 1.34] | 0.612 | 0.87 [0.61, 1.23] | 0.430 | 1.00 [0.52, 1.91] | 0.999 | 2.05 [0.73, 5.74] | 0.173 |
| Main person responsible for household work |  |  |  |  |  |  |  |  |  |  |  |  |
| No | Reference |  | Reference |  | Reference |  | Reference |  | Reference |  | Reference |  |
| Yes | 0.80 [0.52, 1.23] | 0.302 | 0.80 [0.60, 1.06] | 0.126 | **0.51 [0.36, 0.73]** | **<0.001** | 0.91 [0.68, 1.23] | 0.549 | **0.35 [0.20, 0.62]** | **<0.001** | 0.70 [0.32, 1.55] | 0.379 |
| Equal distribution between partners | 0.75 [0.49, 1.16] | 0.200 | 0.76 [0.57, 1.02] | 0.063 | **0.65 [0.47, 0.92]** | **0.013** | 0.79 [0.59, 1.07] | 0.133 | **0.44 [0.26, 0.75]** | **0.003** | **0.21 [0.07, 0.59]** | **0.003** |
| Average domestic stress level (score 0-10) | **1.12 [1.04, 1.21]** | **0.003** | 0.97 [0.92, 1.02] | 0.229 | 1.00 [0.94, 1.07] | 0.930 | **1.07 [1.01, 1.12]** | **0.014** | 1.00 [0.90, 1.12] | 0.959 | 1.00 [0.84, 1.19] | 0.968 |
| I am someone who …  (min 1 – max 7) – mean (SD) |  |  |  |  |  |  |  |  |  |  |  |  |
| …defends his own opinion. | 1.04 [0.90, 1.21] | 0.609 | 1.00 [0.91, 1.10] | 0.995 | **0.89 [0.80, 1.00]** | **0.049** | 1.04 [0.94, 1.15] | 0.428 | 0.90 [0.75, 1.09] | 0.283 | 0.82 [0.63, 1.07] | 0.151 |
| …is independent. | 0.91 [0.79, 1.04] | 0.162 | **0.88 [0.81, 0.97]** | **0.009** | **0.89 [0.80, 0.99]** | **0.035** | **0.87 [0.79, 0.95]** | **0.003** | 1.01 [0.84, 1.20] | 0.940 | 0.77 [0.59, 1.00] | 0.054 |
| …is willing to take risks. | **0.88 [0.78, 0.98]** | **0.026** | **0.89 [0.83, 0.96]** | **0.003** | 0.96 [0.87, 1.05] | 0.379 | 0.94 [0.87, 1.02] | 0.131 | 0.87 [0.75, 1.01] | 0.070 | 1.06 [0.84, 1.33] | 0.622 |
| …is ready to take a stand. | 0.98 [0.83, 1.15] | 0.778 | 1.05 [0.95, 1.17] | 0.341 | 0.96 [0.85, 1.09] | 0.547 | 1.04 [0.93, 1.16] | 0.499 | 0.96 [0.78, 1.18] | 0.684 | 1.32 [0.96, 1.83] | 0.091 |
| …is aggressive. | 1.04 [0.92, 1.16] | 0.556 | 1.02 [0.95, 1.10] | 0.550 | 1.00 [0.91, 1.10] | 0.975 | 1.04 [0.96, 1.12] | 0.331 | 1.05 [0.91, 1.22] | 0.502 | **0.68 [0.51, 0.92]** | **0.012** |

**Supplementary Table 6. Association between biological sex and individual items from the gender score** (derived from principal component analysis) **with cardiometabolic** **(A)** **and health conditions** **(B)**. OR, odds ratio; CI, confidence interval; SD, standard deviation; BMI, body mass index; TIA, transient ischemic attack; COPD, chronic obstructive pulmonary disease; HIV, human immunodeficiency virus. Significant associations are highlighted in bold, while non-significant associations are presented in light grey.

**A**

| **B** | **Immune system disorders (e.g., autoimmune diseases)** | | **Psychiatric diseases** | | **Pulmonary diseases (e.g., asthma, COPD, pulmonary hypertension)** | | **Bone diseases (e.g., osteoporosis)** | | **Infectious diseases (e.g., HIV, hepatitis)** | |
| --- | --- | --- | --- | --- | --- | --- | --- | --- | --- | --- |
|  | **OR [95%CI]** | **p-value** | **OR [95%CI]** | **p-value** | **OR [95%CI]** | **p-value** | **OR [95%CI]** | **p-value** | **OR [95%CI]** | **p-value** |
| Female sex | **1.90 [1.38, 2.61]** | **<0.001** | 1.82 [1.00, 3.34] | 0.052 | **1.39 [1.02, 1.89]** | **0.037** | **2.15 [1.12, 4.14]** | **0.021** | 0.95 [0.52, 1.72] | 0.862 |
| Income |  |  |  |  |  |  |  |  |  |  |
| Earns highest income in household | Reference |  | Reference |  | Reference |  | Reference |  | Reference |  |
| Earns lowest income in household | 0.78 [0.55, 1.11] | 0.167 | 0.82 [0.44, 1.56] | 0.551 | 0.82 [0.58, 1.15] | 0.248 | 1.17 [0.58, 2.37] | 0.66 | **0.31 [0.13, 0.73]** | **0.007** |
| Equal between partners | 0.85 [0.55, 1.31] | 0.462 | 0.95 [0.42, 2.16] | 0.903 | 1.02 [0.67, 1.56] | 0.911 | 1.86 [0.81, 4.25] | 0.143 | 0.84 [0.38, 1.87] | 0.673 |
| Main person responsible for household work |  |  |  |  |  |  |  |  |  |  |
| No | Reference |  | Reference |  | Reference |  | Reference |  | Reference |  |
| Yes | **1.60 [1.04, 2.45]** | **0.033** | 0.59 [0.30, 1.17] | 0.134 | 0.89 [0.62, 1.29] | 0.54 | 2.04 [0.86, 4.83] | 0.105 | 0.80 [0.41, 1.56] | 0.516 |
| Equal distribution between partners | **1.55 [1.00, 2.41]** | **0.049** | 0.62 [0.30, 1.27] | 0.193 | 0.75 [0.51, 1.10] | 0.142 | 1.17 [0.47, 2.95] | 0.732 | 0.62 [0.30, 1.28] | 0.196 |
| Average domestic stress level (score 0-10) | 0.99 [0.93, 1.06] | 0.8 | **1.12 [1.01, 1.25]** | **0.04** | 1.05 [0.99, 1.11] | 0.126 | **0.82 [0.70, 0.95]** | **0.007** | 0.95 [0.83, 1.08] | 0.398 |
| I am someone who …  (min 1 – max 7) – mean (SD) |  |  |  |  |  |  |  |  |  |  |
| …defends his own opinion. | 0.95 [0.84, 1.07] | 0.365 | 1.18 [0.93, 1.49] | 0.182 | 0.95 [0.84, 1.07] | 0.405 | 1.07 [0.84, 1.36] | 0.576 | 0.96 [0.76, 1.21] | 0.72 |
| …is independent. | **0.88 [0.78, 0.99]** | **0.04** | 0.82 [0.66, 1.01] | 0.06 | 0.99 [0.88, 1.11] | 0.813 | **0.77 [0.61, 0.96]** | **0.022** | 0.95 [0.76, 1.19] | 0.678 |
| …is willing to take risks. | 0.94 [0.85, 1.03] | 0.199 | 0.99 [0.83, 1.19] | 0.93 | 1.06 [0.96, 1.16] | 0.271 | 0.92 [0.76, 1.12] | 0.400 | 0.94 [0.79, 1.13] | 0.533 |
| …is ready to take a stand. | 1.06 [0.92, 1.21] | 0.43 | 0.93 [0.73, 1.19] | 0.573 | 1.02 [0.89, 1.16] | 0.821 | 0.94 [0.73, 1.22] | 0.647 | 1.09 [0.84, 1.41] | 0.534 |
| …is aggressive. | **1.10 [1.00, 1.21]** | **0.06** | 0.88 [0.72, 1.07] | 0.19 | **1.15 [1.05, 1.26]** | **0.003** | 0.98 [0.80, 1.21] | 0.871 | 1.17 [0.99, 1.38] | 0.068 |

**Supplementary Table 7. Association between individual items from the gender score** (derived from principal component analysis) **with cardiometabolic** **(A)** **and health conditions** **(B) stratified** **by biological sex**. OR, odds ratio; CI, confidence interval; SD, standard deviation; BMI, body mass index; TIA, transient ischemic attack; COPD, chronic obstructive pulmonary disease; HIV, human immunodeficiency virus. Significant associations are highlighted in bold, while non-significant associations are presented in light grey.

| **A** | **Diabetes** | | **Hypertension** | | **Dyslipidemia** | | **Obesity**  **(BMI > 30kg/m^2^)** | | **Known coronary artery disease** | | **Previous stroke/TIA** | |
| --- | --- | --- | --- | --- | --- | --- | --- | --- | --- | --- | --- | --- |
|  | **Female** | **Male** | **Female** | **Male** | **Female** | **Male** | **Female** | **Male** | **Female** | **Male** | **Female** | **Male** |
| Income |  |  |  |  |  |  |  |  |  |  |  |  |
| Earns highest income in household | Reference | | Reference | | Reference | | Reference | | Reference | | Reference | |
| Earns lowest income in household | 0.91  [0.40, 2.07] | 1.17  [0.72, 1.92] | 0.90  [0.58, 1.41] | **0.51**  **[0.34, 0.78]** | 0.80  [0.45, 1.45] | **0.49**  **[0.30, 0.79]** | 1.04  [0.69, 1.57] | 0.68  [0.45, 1.03] | 2.16 [0.39,11.98] | **0.37**  **[0.17, 0.80]** | 0.29  [0.02, 4.60] | 0.72  [0.25, 2.04] |
| Equal between partners | 0.92  [0.31, 2.71] | 0.74  [0.38, 1.44] | 1.41  [0.80, 2.47] | 0.77  [0.51, 1.17] | 1.49  [0.72, 3.06] | 0.74  [0.45, 1.21] | 0.92  [0.52, 1.62] | 0.87  [0.55, 1.36] | 3.38 [0.48,23.77] | 0.87  [0.43, 1.77] | 4.72 [0.37,60.16] | 1.84  [0.60, 5.66] |
| Main person responsible for household work |  |  |  |  |  |  |  |  |  |  |  |  |
| No | Reference | | Reference | | Reference | | Reference | | Reference | | Reference | |
| Yes | 0.98  [0.31, 3.06] | 0.80  [0.50, 1.30] | 1.43  [0.75, 2.73] | **0.67**  **[0.48, 0.94]** | 0.80  [0.38, 1.66] | **0.43**  **[0.28, 0.65]** | 1.67  [0.88, 3.15] | 0.77  [0.54, 1.10] | 0.71  [0.13, 3.96] | **0.32**  **[0.17, 0.59]** | 0.48  [0.04, 5.88] | 0.68  [0.29, 1.60] |
| Equal distribution between partners | 1.33  [0.40, 4.46] | 0.69  [0.43, 1.12] | 1.05  [0.52, 2.12] | 0.75  [0.54, 1.04] | 0.69  [0.31, 1.57] | **0.68**  **[0.47, 0.98]** | 1.48  [0.75, 2.93] | **0.66**  **[0.46, 0.94]** | 0.71  [0.11, 4.62] | **0.42**  **[0.24, 0.74]** | N/A | **0.25**  **[0.09, 0.70]** |
| Average domestic stress level (score 0-10) | **1.18**  **[1.03, 1.36]** | 1.09  [1.00, 1.20] | 0.98  [0.90, 1.06] | 0.96  [0.89, 1.03] | 1.00  [0.90, 1.11] | 1.00  [0.92, 1.08] | 1.07  [0.99, 1.15] | 1.07  [0.99, 1.14] | 0.96  [0.74, 1.26] | 1.00  [0.89, 1.13] | 0.90  [0.58, 1.39] | 1.01  [0.84, 1.22] |
| I am someone who …  (min 1 – max 7) |  |  |  |  |  |  |  |  |  |  |  |  |
| …defends his own opinion. | 1.30  [0.92, 1.82] | 0.98  [0.83, 1.16] | 0.91  [0.78, 1.07] | 1.04  [0.92, 1.18] | 0.88  [0.72, 1.07] | 0.90  [0.78, 1.03] | 1.06  [0.90, 1.24] | 1.03  [0.90, 1.17] | 1.16  [0.66, 2.05] | 0.87  [0.72, 1.06] | 0.86  [0.37, 1.99] | 0.82  [0.62, 1.10] |
| …is independent. | 0.78  [0.59, 1.04] | 0.94  [0.81, 1.10] | 0.96  [0.82, 1.13] | **0.86**  **[0.76, 0.96]** | 0.89  [0.73, 1.09] | 0.89  [0.79, 1.02] | 0.92  [0.79, 1.07] | **0.84**  **[0.75, 0.95]** | 0.72  [0.44, 1.18] | 1.06  [0.88, 1.29] | 0.58  [0.25, 1.32] | 0.80  [0.60, 1.06] |
| …is willing to take risks. | 0.80  [0.63, 1.02] | 0.91  [0.79, 1.03] | 0.91  [0.80, 1.04] | **0.89**  **[0.81, 0.97]** | 0.90  [0.76, 1.06] | 0.99  [0.89, 1.11] | 0.94  [0.83, 1.07] | 0.95  [0.86, 1.05] | 0.83  [0.54, 1.27] | 0.88  [0.75, 1.04] | 1.13  [0.56, 2.25] | 1.05  [0.83, 1.34] |
| …is ready to take a stand. | 1.13  [0.82, 1.56] | 0.93  [0.77, 1.12] | 0.94  [0.79, 1.12] | 1.12  [0.98, 1.28] | 0.98  [0.78, 1.21] | 0.95  [0.81, 1.11] | 0.94  [0.80, 1.12] | 1.10  [0.95, 1.27] | 1.34  [0.74, 2.44] | 0.91  [0.73, 1.13] | 1.18  [0.47, 2.96] | 1.35  [0.95, 1.91] |
| …is aggressive. | 0.96  [0.74, 1.24] | 1.05  [0.92, 1.20] | 1.00  [0.87, 1.15] | 1.03  [0.94, 1.13] | 0.96  [0.80, 1.15] | 1.01  [0.91, 1.13] | 1.12  [0.99, 1.26] | 0.99  [0.90, 1.09] | 0.99  [0.63, 1.55] | 1.06  [0.90, 1.24] | 0.92  [0.43, 1.96] | **0.65**  **[0.47, 0.90]** |

| **B** | **Immune system disorders (e.g., autoimmune diseases)** | | **Psychiatric diseases** | | **Pulmonary diseases (e.g., asthma, COPD, pulmonary hypertension)** | | **Bone diseases (e.g., osteoporosis)** | | **Infectious diseases (e.g., HIV, hepatitis)** | | **Immune system disorders (e.g., autoimmune diseases)** | |
| --- | --- | --- | --- | --- | --- | --- | --- | --- | --- | --- | --- | --- |
|  | **Female** | **Male** | **Female** | **Male** | **Female** | **Male** | **Female** | **Male** | **Female** | **Male** | **Female** | **Male** |
| Income |  |  |  |  |  |  |  |  |  |  |  |  |
| Earns highest income in household | Reference | | Reference | | Reference | | Reference | | Reference | | Reference | |
| Earns lowest income in household | 0.88  [0.57, 1.37] | 0.69  [0.36, 1.34] | 0.59  [0.27, 1.29] | 1.32  [0.48, 3.65] | 0.27  [0.04, 1.72] | 6.59 [0.91,47.68] | 1.03  [0.66, 1.62] | 0.63  [0.36, 1.13] | 0.99  [0.43, 2.30] | 1.50  [0.36, 6.22] | **0.31**  **[0.09, 0.99]** | 0.36  [0.11, 1.23] |
| Equal between partners | 1.18  [0.68, 2.06] | 0.50  [0.24, 1.06] | 0.49  [0.15, 1.62] | 1.98  [0.63, 6.20] | 2.94 [0.51,16.89] | N/A | 1.11  [0.61, 2.02] | 0.98  [0.54, 1.76] | 1.45  [0.50, 4.19] | 2.82 [0.72,11.05] | 0.35  [0.07, 1.72] | 1.33  [0.52, 3.37] |
| Main person responsible for household work |  |  |  |  |  |  |  |  |  |  |  |  |
| No | Reference | | Reference | | Reference | | Reference | | Reference | | Reference | |
| Yes | **2.21**  **[1.06, 4.61]** | 1.25  [0.70, 2.23] | 0.68  [0.25, 1.81] | 0.49  [0.16, 1.46] | 1.30 [0.14,12.03] | 1.07 [0.08,14.74] | 1.27  [0.67, 2.43] | 0.68  [0.42, 1.12] | 1.96  [0.54, 7.10] | 2.16  [0.61, 7.58] | 2.10 [0.26,17.02] | 0.67  [0.30, 1.48] |
| Equal distribution between partners | 1.86  [0.85, 4.05] | 1.55  [0.89, 2.67] | 0.78  [0.26, 2.33] | 0.51  [0.19, 1.36] | N/A | 1.58 [0.21,11.73] | 0.89  [0.44, 1.82] | 0.72  [0.45, 1.15] | 1.49  [0.38, 5.88] | 0.74  [0.19, 2.85] | 1.61 [0.17,14.82] | 0.54  [0.24, 1.22] |
| Average domestic stress level (score 0-10) | 0.98  [0.91, 1.07] | 1.00  [0.89, 1.11] | 1.08  [0.94, 1.24] | **1.22**  **[1.02, 1.46]** | 1.01  [0.77, 1.32] | 0.90  [0.54, 1.48] | 1.02  [0.94, 1.11] | 1.08  [0.98, 1.18] | **0.80**  **[0.67, 0.95]** | 0.86  [0.65, 1.14] | 0.96  [0.80, 1.15] | 0.94  [0.78, 1.12] |
| I am someone who …  (min 1 – max 7) |  |  |  |  |  |  |  |  |  |  |  |  |
| …defends his own opinion. | 0.96  [0.82, 1.13] | 0.91  [0.75, 1.10] | 1.16  [0.85, 1.59] | 1.26  [0.86, 1.84] | 1.27  [0.67, 2.41] | 0.67  [0.35, 1.26] | 0.95  [0.80, 1.12] | 0.94  [0.79, 1.11] | 1.13  [0.83, 1.55] | 1.00  [0.69, 1.47] | 0.93  [0.63, 1.37] | 0.97  [0.73, 1.29] |
| …is independent. | 0.87  [0.74, 1.02] | 0.91  [0.76, 1.08] | **0.74**  **[0.56, 0.98]** | 0.92  [0.67, 1.28] | 0.51  [0.29, 0.90] | 1.04  [0.52, 2.11] | 1.11  [0.92, 1.33] | 0.91  [0.77, 1.07] | **0.65**  **[0.49, 0.87]** | 0.99  [0.68, 1.44] | 1.23  [0.81, 1.88] | 0.86  [0.66, 1.12] |
| …is willing to take risks. | 0.93  [0.82, 1.06] | 0.95  [0.82, 1.11] | 1.01  [0.79, 1.28] | 0.96  [0.72, 1.27] | 1.12  [0.71, 1.79] | 0.98  [0.54, 1.78] | 1.04  [0.91, 1.19] | 1.08  [0.94, 1.24] | 0.89  [0.70, 1.14] | 0.98  [0.71, 1.37] | 0.98  [0.73, 1.32] | 0.93  [0.75, 1.16] |
| …is ready to take a stand. | 1.09  [0.91, 1.30] | 1.01  [0.81, 1.24] | 1.02  [0.74, 1.38] | 0.82  [0.56, 1.21] | 1.10  [0.60, 2.04] | 1.40  [0.61, 3.24] | 1.09  [0.90, 1.32] | 0.94  [0.78, 1.14] | 1.14  [0.81, 1.60] | 0.68  [0.45, 1.03] | 0.87  [0.58, 1.31] | 1.22  [0.88, 1.71] |
| …is aggressive. | 1.06  [0.93, 1.20] | 1.15  [0.99, 1.33] | 0.86  [0.66, 1.13] | 0.88  [0.65, 1.19] | 1.10  [0.70, 1.74] | 1.08  [0.58, 2.02] | **1.15**  **[1.01, 1.31]** | **1.15**  **[1.01, 1.30]** | 1.01  [0.79, 1.30] | 0.92  [0.64, 1.32] | **1.36**  **[1.03, 1.78]** | 1.08  [0.88, 1.34] |


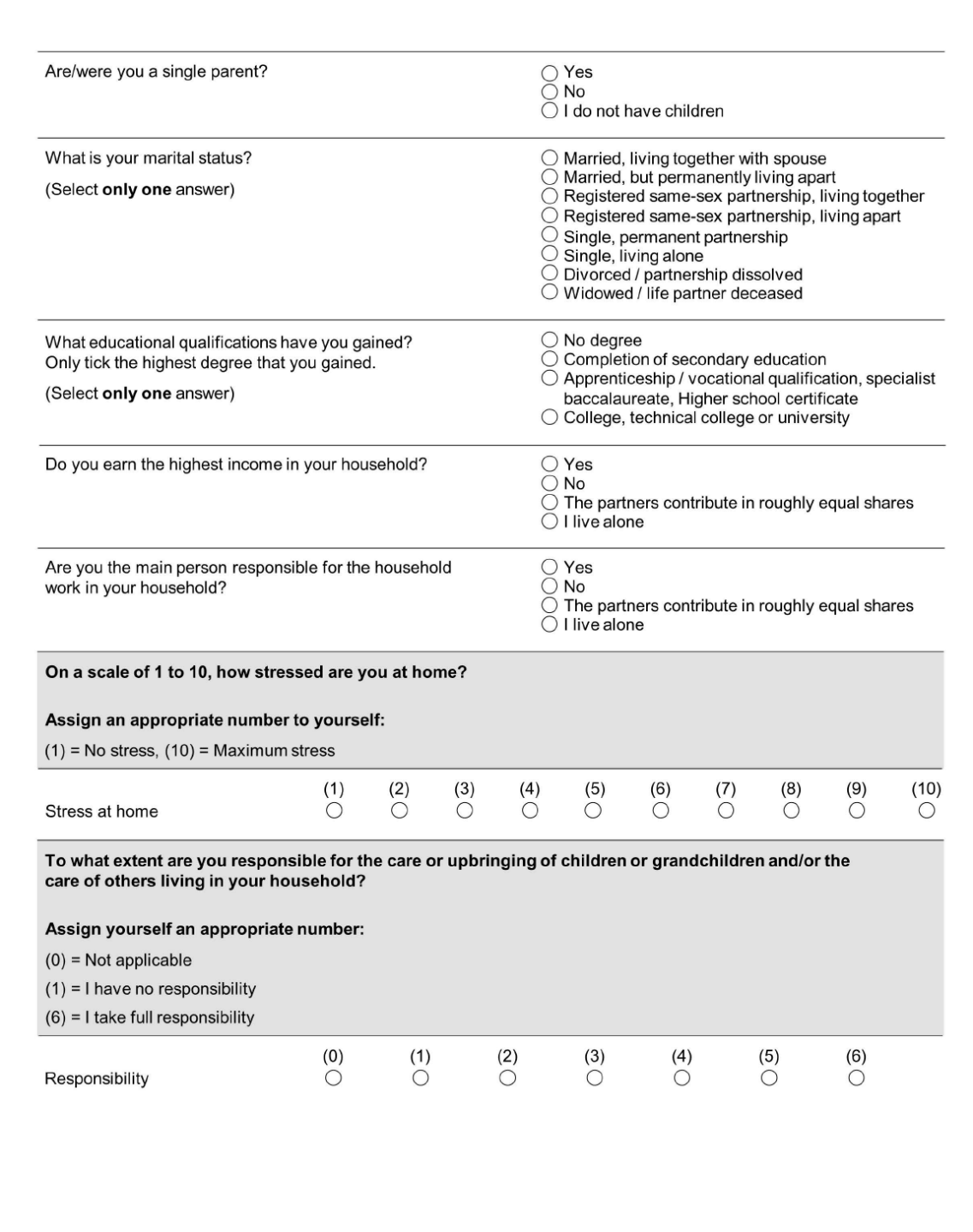
**Supplementary Figure 1:** **Gender-related questions included in the questionnaire.**


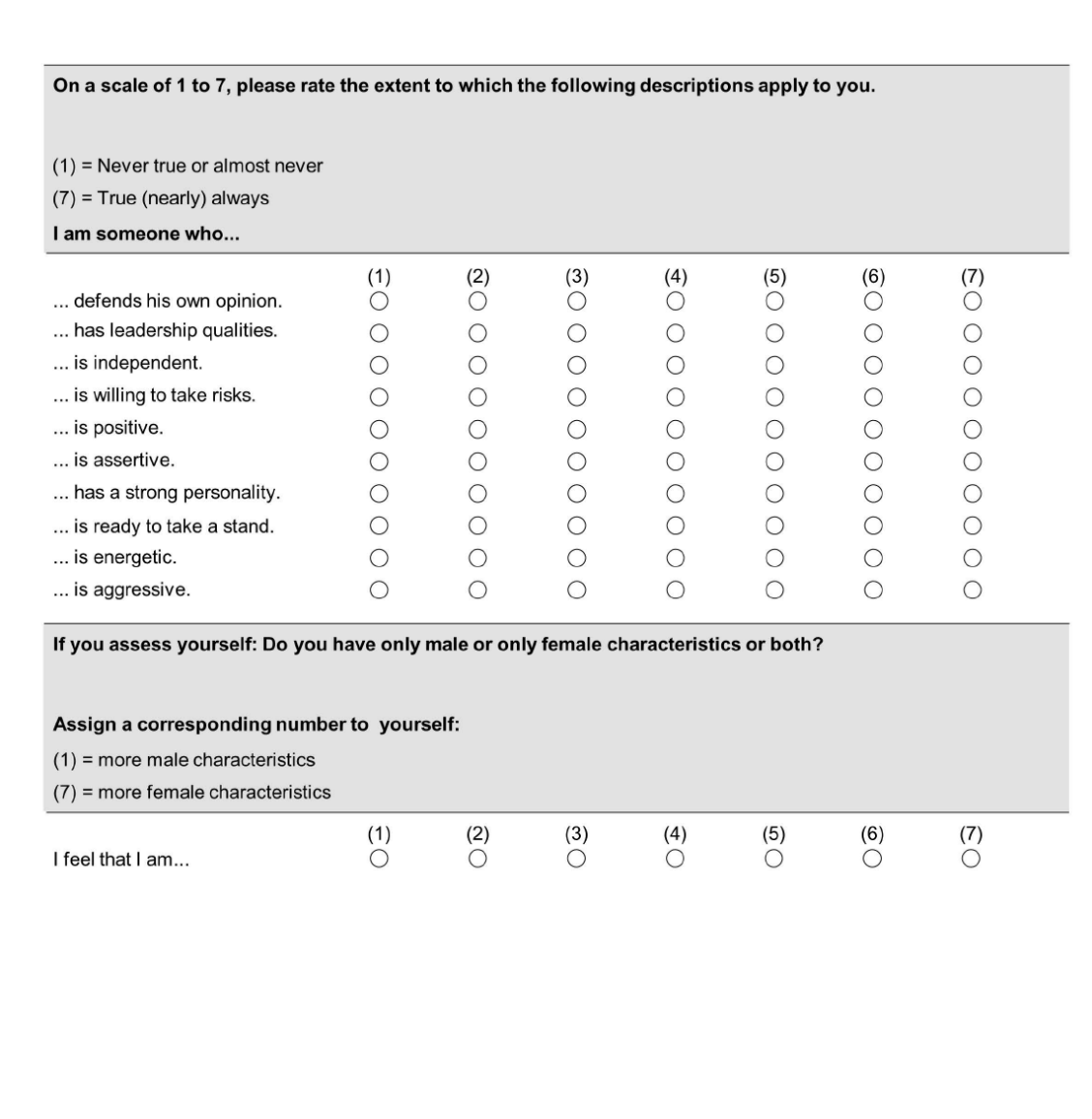


**Supplementary Figure 2: Flowchart providing an overview of the estimation and validation data sets.**

**
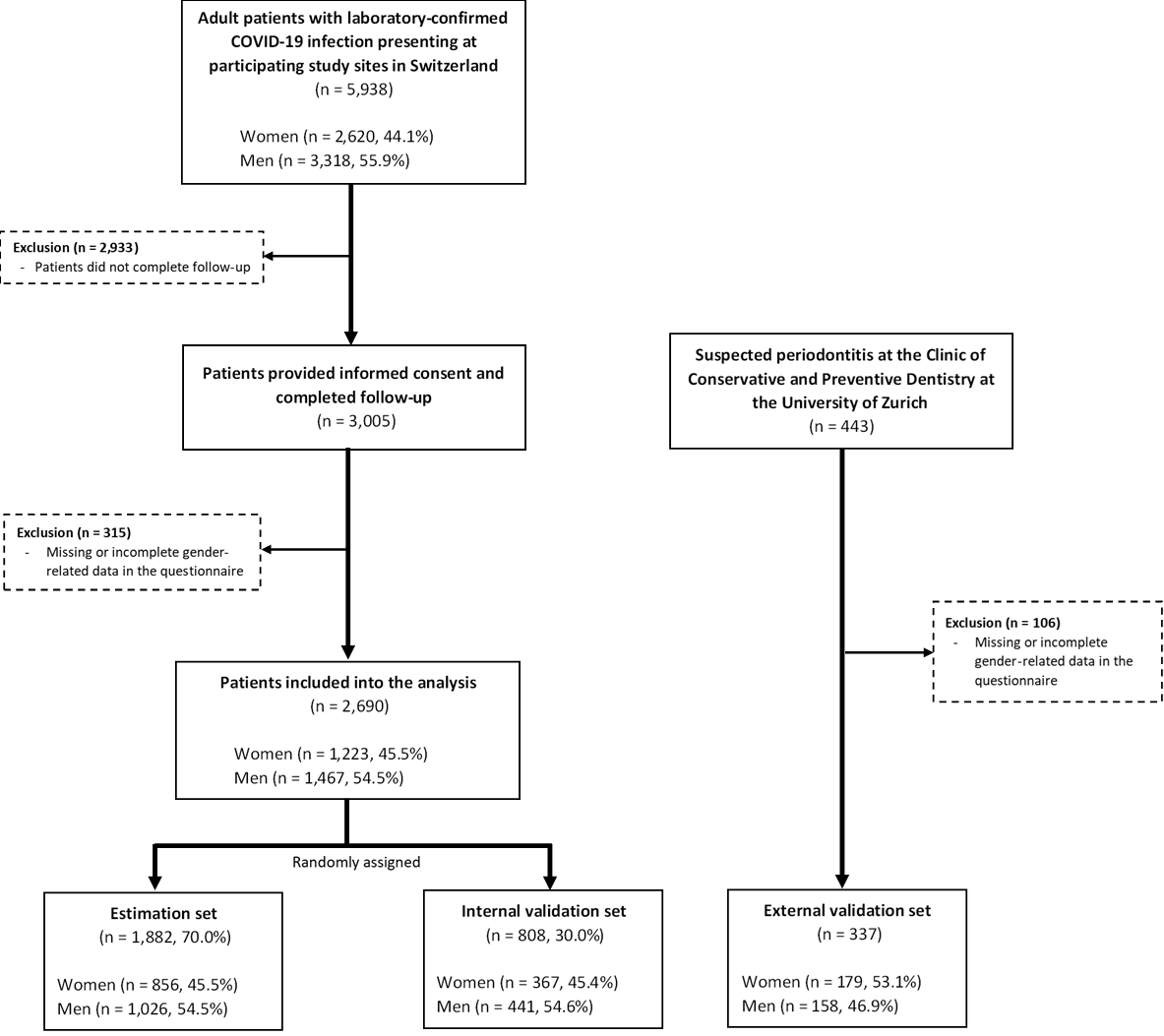
**

**Supplementary Figure 3:** Distribution of the gender score by biological sex across the estimation, internal validation, and external validation cohorts. Violin plots display the distribution of gender scores within each dataset, with embedded boxplots showing medians and interquartile ranges.

**
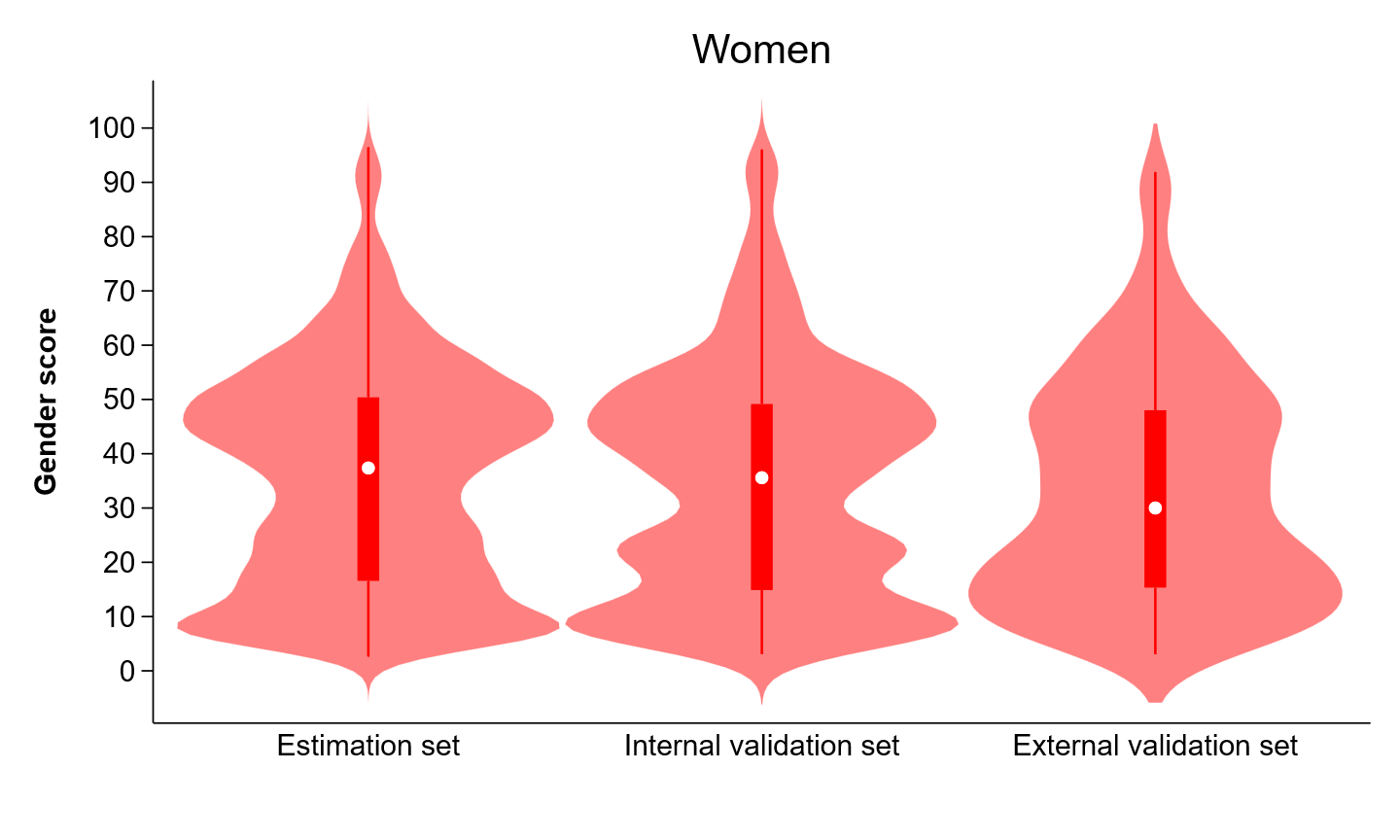
**

**
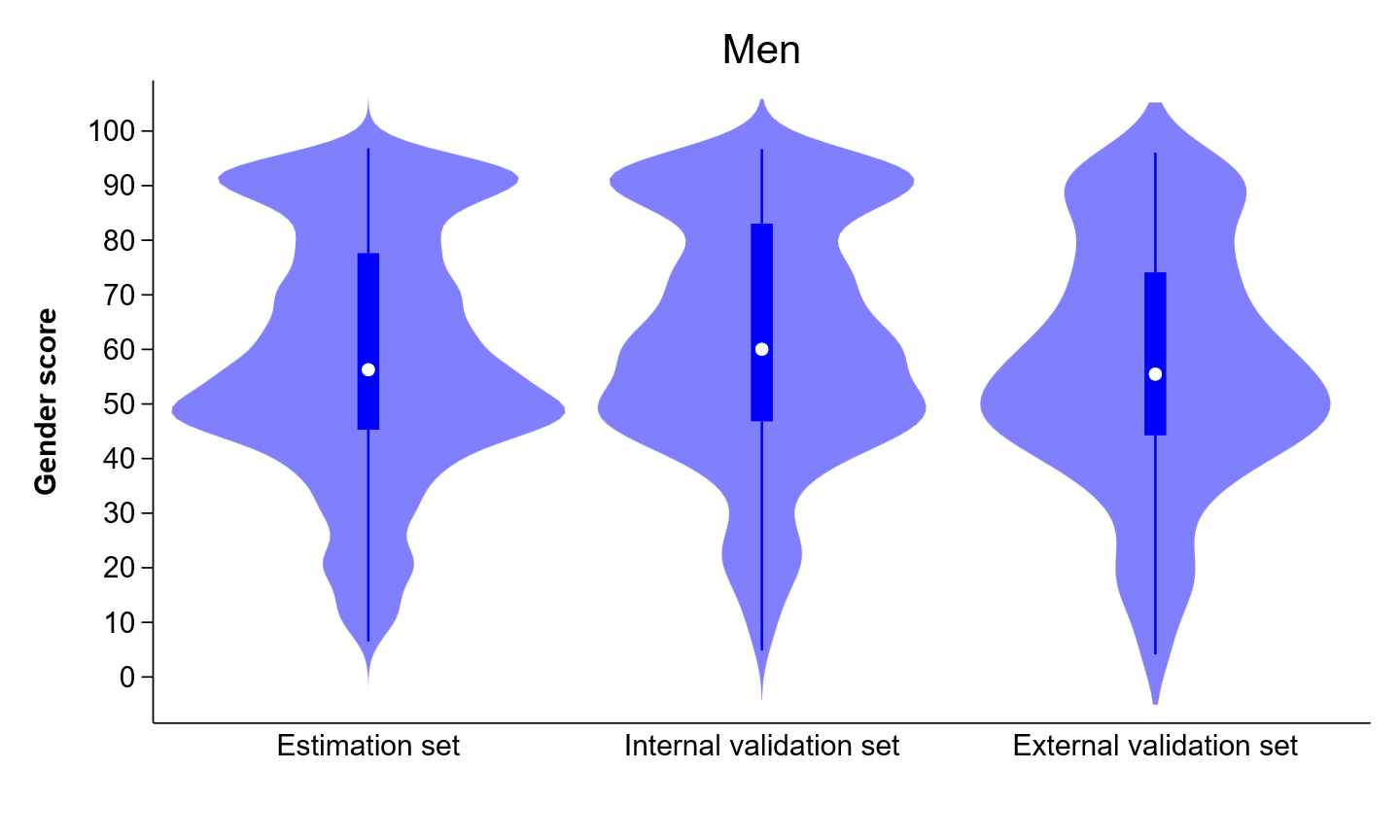
**
